# A comprehensive analysis of minimally differentially methylated regions common to pediatric and adult solid tumors

**DOI:** 10.1038/s41698-024-00590-1

**Published:** 2024-06-01

**Authors:** David N. Buckley, Ben Yi Tew, Chris Gooden, Bodour Salhia

**Affiliations:** 1 Department of Translational Genomics, Keck School of Medicine, University of Southern California, Los Angeles, CA USA; 2 Norris Comprehensive Cancer Center, University of Southern California, Los Angeles, CA USA

**Keywords:** Cancer genomics, Paediatric cancer

## Abstract

Cancer is the second most common cause of death in children aged 1–14 years in the United States, with 11,000 new cases and 1200 deaths annually. Pediatric cancers typically have lower mutational burden compared to adult-onset cancers, however, the epigenomes in pediatric cancer are highly altered, with widespread DNA methylation changes. The rarity of pediatric cancers poses a significant challenge to developing cancer-type specific biomarkers for diagnosis, prognosis, or treatment monitoring. In the current study, we explored the potential of a DNA methylation profile common across various pediatric cancers. To do this, we conducted whole genome bisulfite sequencing (WGBS) on 31 recurrent pediatric tumor tissues, 13 normal tissues, and 20 plasma cell-free (cf)DNA samples, representing 11 different pediatric cancer types. We defined minimal focal regions that were differentially methylated across samples in the multiple cancer types which we termed minimally differentially methylated regions (mDMRs). These methylation changes were also observed in 506 pediatric and 5691 adult cancer samples accessed from publicly available databases, and in 44 pediatric cancer samples we analyzed using a targeted hybridization probe capture assay. Finally, we found that these methylation changes were detectable in cfDNA and could serve as potential cfDNA methylation biomarkers for early detection or minimal residual disease.

## Introduction

Cancer is the second leading cause of death in children aged 1–14 years in the United States, with approximately 11,000 new cases and 1200 deaths annually^1^. The 5-year overall survival rate for pediatric cancer has risen dramatically in recent years, from 58% in 1975 to just under 85% in 2020^2^. However, neuroblastoma – the third most common form of pediatric cancer after leukemia and central nervous system (CNS) tumors – has a 5-year overall survival rate of 75% which plummets to 20% after the first disease recurrence^3^. The improvement in overall survival for metastatic pediatric cancers has been mixed over the last few decades, with neuroblastoma showing improvement in prognosis due to treatment advances, while others like rhabdomyosarcoma and Ewing sarcoma have had minimal improvement^4^. A key factor limiting progress in this is the rarity of many of these cancers, which limits research opportunities and hampers clinical trials.

The rarity of pediatric cancers poses a significant challenge to developing cancer-type specific diagnostic, prognostic or predictive biomarkers. In this context, it is appealing to consider a common molecular signature across multiple cancer types, an approach that has also shown promise in multi-cancer early detection testing^5,6^. Specifically, we aimed to identify DNA methylation changes common to multiple pediatric cancers. Compared to adult-onset cancer, pediatric cancer genomes are characteristically ‘*quiet’*, with a low mutational burden^7^, while the epigenome of many pediatric cancers appears ‘*loud’*, with many driver mutations occurring in chromatin modifiers, alongside widespread DNA methylation and histone mark changes^8^. DNA methylation changes are abundant in cancer and are among the earliest aberrations to occur in tumorigenesis^9^.

In the current study, we aimed to identify DNA methylation changes common to multiple non-CNS pediatric solid cancers. We performed whole genome bisulfite sequencing (WGBS) of pediatric cancers, including 31 tumor tissue, 13 normal tissue, and 20 plasma cfDNA samples from 27 individuals, representing 11 different pediatric cancer subtypes. By integrating data across tumor types, we identified the minimal focal regions that were differentially methylated in the most number of samples across cancer types, which we termed minimally differentially methylated regions (mDMRs). We also found these mDMRs in 506 pediatric cancer samples from 4 cancer types, sourced from ‘Therapeutically Applicable Research to Generate Effective Treatments’ (TARGET), and 5691 adult cancer samples, sourced from ‘The Cancer Genome Atlas’ (TCGA), from 14 cancer types, and in several CNS tumor types sourced from Capper et al.^10^. We performed further validation using a targeted hybridization probe capture assay in an independent set of 44 pediatric cancer tissue samples from 6 tumor types. Finally, we found that these methylation changes were detectable in cell-free (cf)DNA and could serve as potential cfDNA methylation biomarkers.

The identification of such a DNA methylation panel in pediatric cancers could potentially provide the basis for cfDNA methylation liquid biopsy for early detection, treatment response, or minimal residual disease (MRD). Thus, our study’s endeavor to uncover such a methylation pattern has both scientific and clinical value, aiming to translate these molecular insights into tangible advances for both pediatric and adult cancer.

## Results

### Analysis of differential DNA methylation patterns in pediatric cancers

We performed WGBS on 31 tumor and 13 patient-matched adjacent normal tissue samples representing 11 different pediatric cancer types (Table 1). First, we performed differential methylation analysis to find differentially methylated regions (DMRs); tumor samples were compared with their patient-matched adjacent normal sample where possible. For tumor samples without a matched normal, a pool of normal samples from other patients with the same diagnosis was used. Differential DNA methylation analysis revealed a variable number of DMRs both within and between tumor types (SF1 A/B, Supplementary Table S1). On average, 74% of DMRs across all tumors were hypomethylated compared to 26% hypermethylated DMRs. However, malignant rhabdoid tumors (MRT) displayed a hypermethylator phenotype where 90% of DMRs were hypermethylated (SF2A). Global hypermethylation in MRT was also observed in 68 TARGET samples (SF2B).

**Table 1:** A: Sample summary. B: Patient summary

(A)					
Sample.ID	Patient.ID	Diagnosis.Abbreviation	Diagnosis	Sample.Type	Resection.Location
P01-010-P-1	P01-010	NBL	Neuroblastoma	PLASMA	Peripheral Blood
P01-010-N-1	P01-010	NBL	Neuroblastoma	NORMAL_TISSUE	Adjacent tissue normal
P01-010-T-1	P01-010	NBL	Neuroblastoma	CANCER_TISSUE	Unspecified
P01-012-P-1	P01-012	OS	Osteosarcoma	PLASMA	Peripheral Blood
P01-012-N-1	P01-012	OS	Osteosarcoma	NORMAL_TISSUE	Adjacent tissue normal
P01-012-T-1	P01-012	OS	Osteosarcoma	CANCER_TISSUE	Lung
P01-014-P-1	P01-014	AE	Anaplastic Ependymoma	PLASMA	Peripheral Blood
P01-014-T-1	P01-014	AE	Anaplastic Ependymoma	CANCER_TISSUE	Unspecified
P01-015-N-1	P01-015	FHC	Fibrolamellar Hepatocellular Carcinoma	NORMAL_TISSUE	Adjacent tissue normal
P01-015-T-1	P01-015	FHC	Fibrolamellar Hepatocellular Carcinoma	CANCER_TISSUE	Unspecified
P01-016-N-1	P01-016	OS	Osteosarcoma	NORMAL_TISSUE	Adjacent tissue normal
P01-016-T-1	P01-016	OS	Osteosarcoma	CANCER_TISSUE	Lung
P01-017-P-1	P01-017	NBL	Neuroblastoma	PLASMA	Peripheral Blood
P01-017-T-1	P01-017	NBL	Neuroblastoma	CANCER_TISSUE	Unspecified
P01-018-P-1	P01-018	NBL	Neuroblastoma	PLASMA	Peripheral Blood
P01-018-T-1	P01-018	NBL	Neuroblastoma	CANCER_TISSUE	Abdomen
P01-019-N-1	P01-019	MRT	Malignant Rhabdoid Tumor	NORMAL_TISSUE	Adjacent tissue normal
P01-019-T-1	P01-019	MRT	Malignant Rhabdoid Tumor	CANCER_TISSUE	Abdomen
P01-019-T-2	P01-019	MRT	Malignant Rhabdoid Tumor	CANCER_TISSUE	Abdomen
P01-020-AC3-P-1	P01-020	HB	Hepatoblastoma	PLASMA	Peripheral Blood
P01-020-P-1	P01-020	HB	Hepatoblastoma	PLASMA	Peripheral Blood
P01-020-N-1	P01-020	HB	Hepatoblastoma	NORMAL_TISSUE	Adjacent tissue normal
P01-020-T-1	P01-020	HB	Hepatoblastoma	CANCER_TISSUE	Unspecified
P01-020-T-2	P01-020	HB	Hepatoblastoma	CANCER_TISSUE	Lung
P01-021-P-1	P01-021	HB	Hepatoblastoma	PLASMA	Peripheral Blood
P01-021-N-1	P01-021	HB	Hepatoblastoma	NORMAL_TISSUE	Adjacent tissue normal
P01-021-T-1	P01-021	HB	Hepatoblastoma	CANCER_TISSUE	Lung
P01-022-P-1	P01-022	ERMS	Embryonal rhabdomyosarcoma	PLASMA	Peripheral Blood
P01-022-N-1	P01-022	ERMS	Embryonal rhabdomyosarcoma	NORMAL_TISSUE	Adjacent tissue normal
P01-022-T-1	P01-022	ERMS	Embryonal rhabdomyosarcoma	CANCER_TISSUE	Omentum
P01-022-T-2	P01-022	ERMS	Embryonal rhabdomyosarcoma	CANCER_TISSUE	Diaphram
P01-022-T-3	P01-022	ERMS	Embryonal rhabdomyosarcoma	CANCER_TISSUE	Sinus venous mass
P01-022-T-4	P01-022	ERMS	Embryonal rhabdomyosarcoma	CANCER_TISSUE	Pelvic mass
P01-022-T-5	P01-022	ERMS	Embryonal rhabdomyosarcoma	CANCER_TISSUE	Sigmoid colon
P01-023-P-1	P01-023	NBL	Neuroblastoma	PLASMA	Peripheral Blood
P01-023-T-1	P01-023	NBL	Neuroblastoma	CANCER_TISSUE	Mediastinal mass
P01-024-P-1	P01-024	WT	Wilm’s Tumor	PLASMA	Peripheral Blood
P01-024-N-1	P01-024	WT	Wilm’s Tumor	NORMAL_TISSUE	Adjacent tissue normal
P01-024-T-1	P01-024	WT	Wilm’s Tumor	CANCER_TISSUE	Unspecified
P01-025-P-1	P01-025	FHC	Fibrolamellar Hepatocellular Carcinoma	PLASMA	Peripheral Blood
P01-025-T-1	P01-025	FHC	Fibrolamellar Hepatocellular Carcinoma	CANCER_TISSUE	Unspecified
P01-026-P-1	P01-026	NBL	Neuroblastoma	PLASMA	Peripheral Blood
P01-026-T-1	P01-026	NBL	Neuroblastoma	CANCER_TISSUE	Adrenal Gland
P01-027-T-1	P01-027	Hodgkins	Hodgkin’s Lymphoma	CANCER_TISSUE	Unspecified
P01-028-P-1	P01-028	Teratoma	Immature teratoma	PLASMA	Peripheral Blood
P01-028-N-1	P01-028	Teratoma	Immature teratoma	NORMAL_TISSUE	Adjacent tissue normal
P01-028-T-1	P01-028	Teratoma	Immature teratoma	CANCER_TISSUE	Cul De Sac
P01-028-T-2	P01-028	Teratoma	Immature teratoma	CANCER_TISSUE	Ileum
P01-029-P-1	P01-029	OS	Osteosarcoma	PLASMA	Peripheral Blood
P01-029-N-1	P01-029	OS	Osteosarcoma	NORMAL_TISSUE	Adjacent tissue normal
P01-029-T-1	P01-029	OS	Osteosarcoma	CANCER_TISSUE	Retroperitoneum
P01-030-N-1	P01-030	OS	Osteosarcoma	NORMAL_TISSUE	Adjacent tissue normal
P01-030-T-1	P01-030	OS	Osteosarcoma	CANCER_TISSUE	Lung
P01-033-T-1	P01-033	NBL	Neuroblastoma	CANCER_TISSUE	Retroperitoneum
P01-035-T-1	P01-035	DSRCT	Desmoplastic Small Round Cell Tumor	CANCER_TISSUE	Gastric lesion
P01-036-P-1	P01-036	NBL	Neuroblastoma	PLASMA	Peripheral Blood
P01-036-T-2	P01-036	NBL	Neuroblastoma	CANCER_TISSUE	Abdomen
P01-037-P-1	P01-037	NBL	Neuroblastoma	PLASMA	Peripheral Blood
P01-037-N-1	P01-037	NBL	Neuroblastoma	NORMAL_TISSUE	Adjacent tissue normal
P01-037-T-1	P01-037	NBL	Neuroblastoma	CANCER_TISSUE	Unspecified
P01-040-T-1	P01-040	NBL	Neuroblastoma	CANCER_TISSUE	Unspecified
P01-0813-N	P01-0813	Healthy donor	Healthy donor	PLASMA	Peripheral Blood
P01-2013-N	P01-2013	Healthy donor	Healthy donor	PLASMA	Peripheral Blood
P01-2714-N	P01-2714	Healthy donor	Healthy donor	PLASMA	Peripheral Blood
SAL-2	SAL	Healthy donor	Healthy donor	PLASMA	Peripheral Blood
SAL-4	SAL	Healthy donor	Healthy donor	PLASMA	Peripheral Blood
SAL-5	SAL	Healthy donor	Healthy donor	PLASMA	Peripheral Blood
SAL-7	SAL	Healthy donor	Healthy donor	PLASMA	Peripheral Blood
SAL-8	SAL	Healthy donor	Healthy donor	PLASMA	Peripheral Blood
BAR-1	BAR	Healthy donor	Healthy donor	PLASMA	Peripheral Blood
BAR-2	BAR	Healthy donor	Healthy donor	PLASMA	Peripheral Blood
BAR-3	BAR	Healthy donor	Healthy donor	PLASMA	Peripheral Blood
BAR-5	BAR	Healthy donor	Healthy donor	PLASMA	Peripheral Blood
HP-1	HP-1	Healthy donor	Healthy donor	PLASMA	Peripheral Blood
HP-2	HP-2	Healthy donor	Healthy donor	PLASMA	Peripheral Blood
HP-AA	HP-AA	Healthy donor	Healthy donor	PLASMA	Peripheral Blood

(B)								
Patient ID	Sex	Age	Tumor samples	Normal samples	Plasma samples	Diagnosis	Diagnosis abbreviation	Source
P01-010	F	35	1	1	1	Neuroblastoma	NBL	POETIC
P01-012	M	16	1	1	1	Osteosarcoma	OS	POETIC
P01-014	F	10	1	0	1	Anaplastic Ependymoma	AE	POETIC
P01-015	M	15	1	1	0	Fibrolamellar Hepatocellular Carcinoma	FHC	POETIC
P01-016	M	19	1	1	0	Osteosarcoma	OS	POETIC
P01-017	M	5	1	0	1	Neuroblastoma	NBL	POETIC
P01-018	M	34	1	0	1	Neuroblastoma	NBL	POETIC
P01-019	F	16	2	1	0	Malignant Rhabdoid Tumor	MRT	POETIC
P01-020	M	2	2	1	2	Hepatoblastoma	HB	POETIC
P01-021	M	3	1	1	1	Hepatoblastoma	HB	POETIC
P01-022	F	8	5	1	1	Embryonal rhabdomyosarcoma	ERMS	POETIC
P01-023	M	3	1	0	1	Neuroblastoma	NBL	POETIC
P01-024	M	1.5	1	1	1	Wilm’s Tumor	WT	POETIC
P01-025	M	16	1	0	1	Fibrolamellar Hepatocellular Carcinoma	FHC	POETIC
P01-026	F	2	1	0	1	Neuroblastoma	NBL	POETIC
P01-027	F	19	1	0	0	Hodgkin’s Lymphoma	Hodgkins	POETIC
P01-028	F	14	2	1	1	Immature teratoma	Teratoma	POETIC
P01-029	M	14	1	1	1	Osteosarcoma	OS	POETIC
P01-030	F	19	1	1	0	Osteosarcoma	OS	POETIC
P01-033	M	7	1	0	0	Neuroblastoma	NBL	POETIC
P01-035	M	14	1	0	0	Desmoplastic Small Round Cell Tumor	DSRCT	POETIC
P01-036	M	12	1	0	1	Neuroblastoma	NBL	POETIC
P01-037	F	4	1	1	1	Neuroblastoma	NBL	POETIC
P01-040	F	10	1	0	0	Neuroblastoma	NBL	POETIC
P01-0813	F		0	0	1	Healthy		Conversant
P01-2013	F		0	0	1	Healthy		Conversant
P01-2714	F		0	0	1	Healthy		Conversant
SAL	F		0	0	5	Healthy		Salhia Lab
BAR	F		0	0	4	Healthy		Salhia Lab
HP-1^a^	F		0	0	1	Healthy		Salhia Lab
HP-2^a^	F		0	0	1	Healthy		Salhia Lab
HP-AA^a^	F		0	0	1	Healthy		Salhia Lab
TOTAL:			31	13	32			

(A) All unique samples in the study used for WGBS. The diagnosis for each patient is indicated, along with the sample type and resection location where applicable.

(B) All unique cases in the study with demographic information, diagnosis, and sample source. Number of tumor tissue, normal tissue and plasma samples analyzed per patient is indicated.

^a^HP-1, HP-2, and HP-AA are cfDNA pools comprised of 9, 10, and 20 healthy patients’ DNA, respectively.

**Fig. 1:**
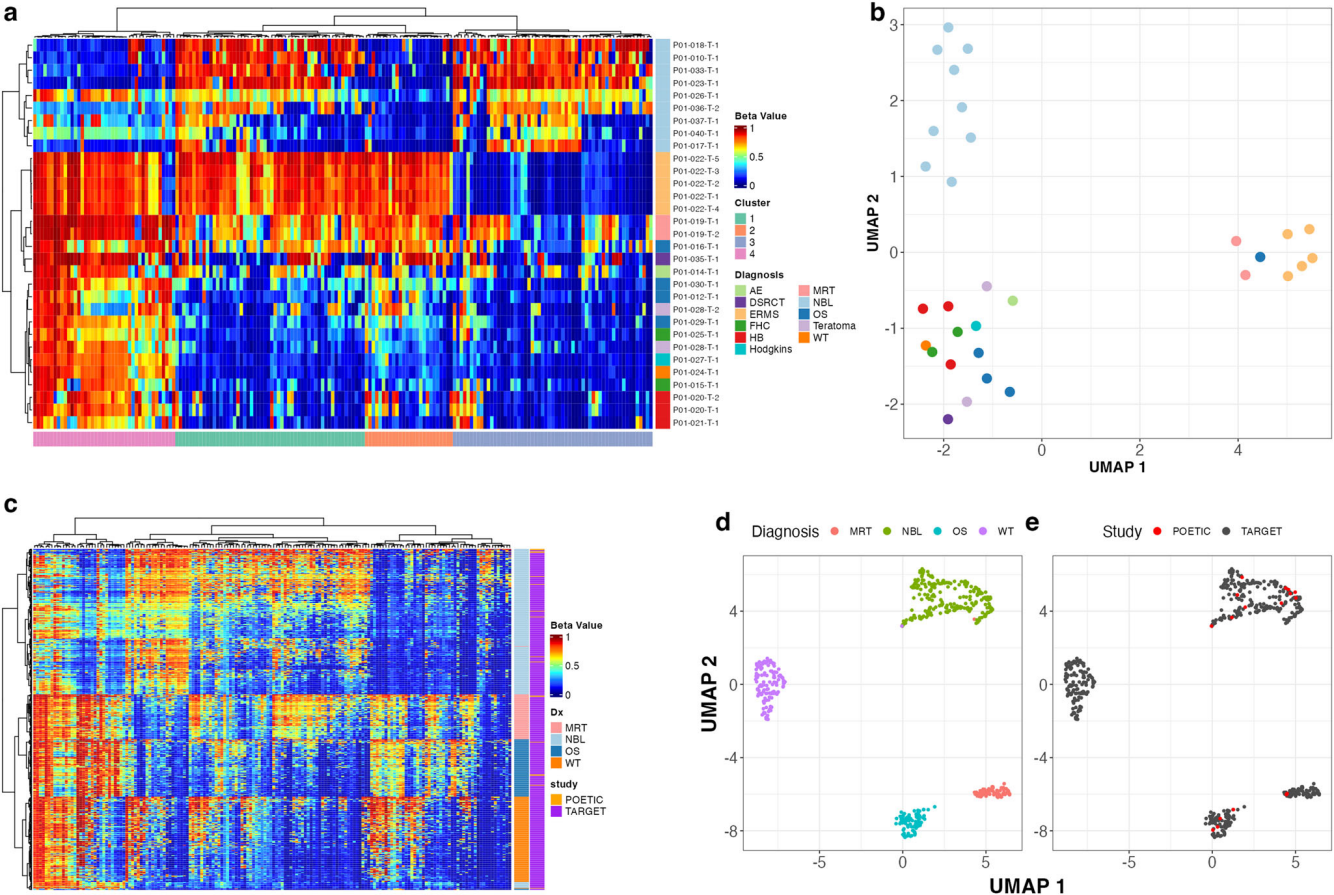
Analysis of differential DNA methylation patterns in pediatric cancers. **a** Hierarchical clustering of beta values across 183 highly variable DMRs in 31 tumor samples. Row annotation bar denotes diagnosis; column annotation bar denotes DMR cluster as determined by k-means clustering. **b** Uniform manifold approximation projection (UMAP) per sample based 183 most variable DMRs as in in **c**. **c** Heatmap of beta values of 166/183 regions in C (17 regions from **A** were dropped due to limitations of 450 K array) from 506 TARGET samples and 17 POETIC samples. **d** UMAP of TARGET & POETIC samples across regions in **C** by diagnosis and **e** source.

Hierarchical clustering using the 2.5% most variable statistically significant DMR calls (183 unique regions after analyzing each tumor/normal comparison) showed separation by tumor type and identified 4 distinct DMR clusters (Fig. 1a, b, SF3). These DMRs were predominantly located at CpG islands (*n* = 151), with the remaining DMRs located in CpG shores (*n* = 6), shelves (*n* = 4), and open sea (*n* = 22). Ingenuity pathway analysis (IPA) of Cluster 1 genes found enrichment for netrin signaling and GABA receptor signaling (Supplementary Table S2). Cluster 2 showed enrichment for embryonic stem cell differentiation and sonic hedgehog signaling (Supplementary Table S3). Cluster 3, (Fig. 1a, Supplementary Table S4, SF3), which was predominately hypermethylated in neuroblastoma (NBL), was associated with ERK/MAPK signaling. Cluster 4 was hypomethylated in neuroblastoma and hypermethylated in all other tumor types. Genes associated with this cluster included several tumor suppressors (BRCA^11^, KANK1^12^, ASB3^13^, NBAT1^14^, PIP4K2A^15^, NFATC1^16^, and ZNRF3^17,18^) and oncogenes (HOXA3^19^ and HOXB-AS3^20^). IPA identified genes with DMRs in this cluster to be associated with PI3K signaling, DNA double-strand break repair, and G2/M checkpoint regulation (Supplementary Table S5).

Methylation beta values were also extracted from 506 TARGET cancer samples (Supplementary Table S6) across 166 of the 183 highly variable DMRs which overlapped at least one probe on the HM450 methylation array. Analysis of TARGET data also demonstrated strong separation by tumor type (Fig. 1c, d). POETIC samples clustered with TARGET samples according to tumor type as seen by hierarchical clustering and UMAP analyses (Fig. 1e). It is also important to note that TARGET samples were predominantly collected from the primary tumors (Supplementary Table S6), while POETIC cases were all collected from patients with recurrent or metastatic disease, indicating that methylation profiles of recurrent tumors are more similar to primary tumors then they are different. The co-clustering of POETIC recurrent samples with TARGET samples was also observed when each tumor type was analyzed separately (data not shown).

### DNA methylation alterations shared across tumor types

**Fig. 2:**
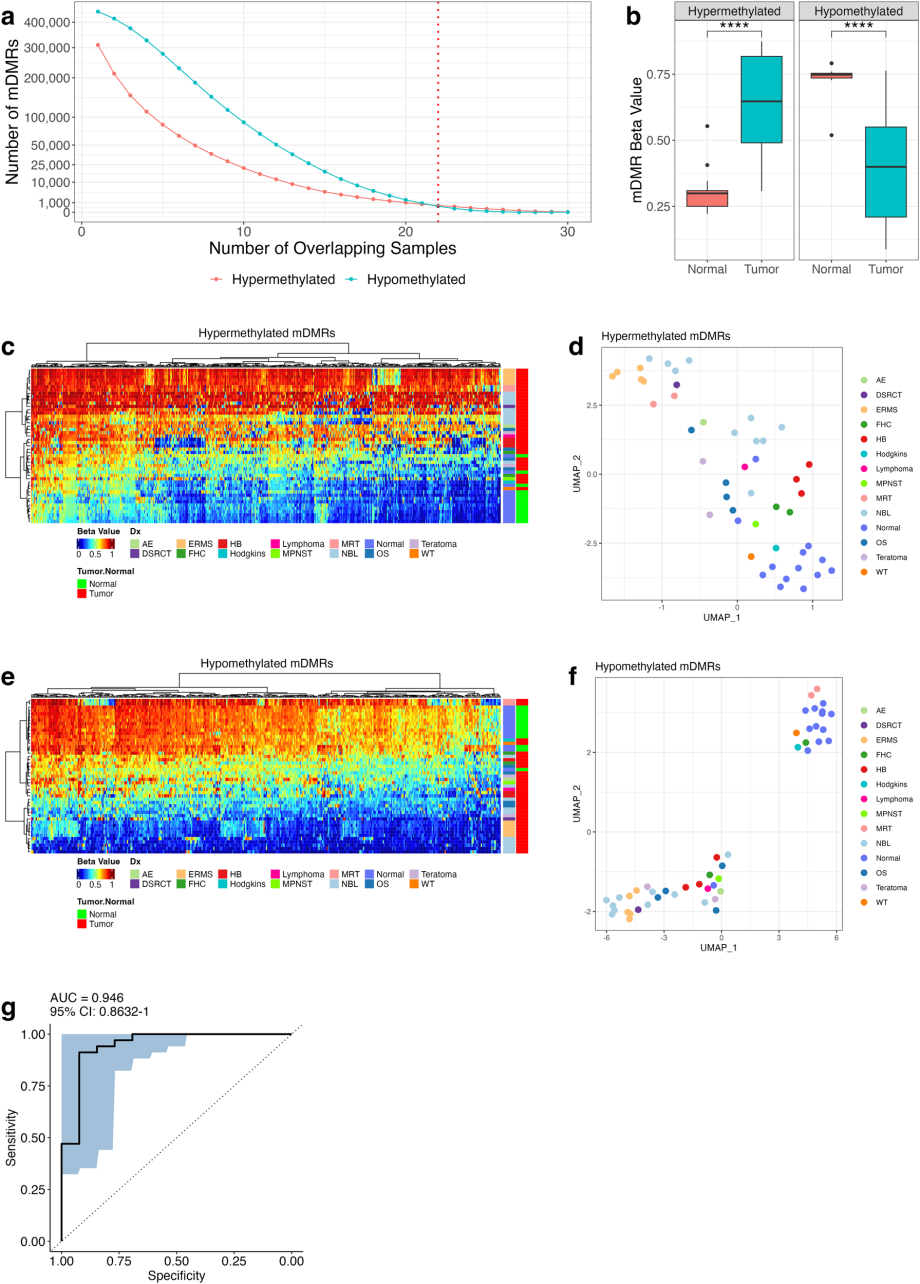
WGBS analysis reveals pan pediatric cancer DNA methylation panel. **a** Number of overlapping mDMRs (y-axis) in a given number of samples (x-axis) by directionality (light blue = hypomethylated regions; red = hypermethylated regions). Red dotted vertical line indicates sample count cutoff (22 samples or greater; 70% of tumor samples) which resulted in 402 hypomethylated and 503 hypermethylated mDMRs. **b** Box plots of average beta values for hypo and hypermethylated mDMRs. Tumor/normal comparisons are statistically significant (*p* < 0.0001; Wilcoxon rank sum test). **c** Heatmap and **d** UMAP generated from beta values across all hypomethylated mDMR regions in all POETIC samples. **e** Heatmap and **f** UMAP generated from beta values across all hypermethylated mDMR regions in all POETIC samples. **g** Receiver operating characteristic (ROC) curve of random forest classifier model constructed using mDMRs. ROC curve annotated with area under the curve (AUC). Significance annotation: ns: *p* > 0.05, *p ≤ 0.05, **p ≤ 0.01, ***p ≤ 0.001, ****p ≤ 0.0001.

We identified minimally differentially methylated regions (mDMRs), as subregions of each DMR that were shared (in the same direction; hypo- or hyper-methylated) across multiple samples and therefore cancer types. Briefly, each CpG site was scored based on the number of samples with a DMR call which included that CpG (separately for hypomethylated and hypermethylated DMR calls). Clusters of adjacent CpG sites, each having a DMR present in at least N samples, were merged to create contiguous mDMR regions. The number of shared mDMRs decreased as N increased, for both hypo and hypermethylated regions (Fig. 2a), but it was possible to identify a set of mDMRs shared between at least 70% (*n* = 22 of 31) of samples. This subset of mDMRs included 402 hypomethylated regions, with a median width of 276 bp, and 503 hypermethylated regions (total = 905, Fig. 2a), with a median width of 230 bp. Mean beta values across hypomethylated mDMRs were 0.406 in tumor tissue compared with 0.732 in normal tissue. Beta values in hypermethylated mDMRs were 0.643 in tumor tissue compared with 0.308 in normal tissues (Fig. 2b). Hypermethylated mDMRs strongly discriminated multiple tumor types from normal tissues by hierarchical clustering (Fig. 2c) and UMAP (Fig. 2d). We observed similar results in hypomethylated mDMRs (Fig. 2e, f).

**Fig. 3:**
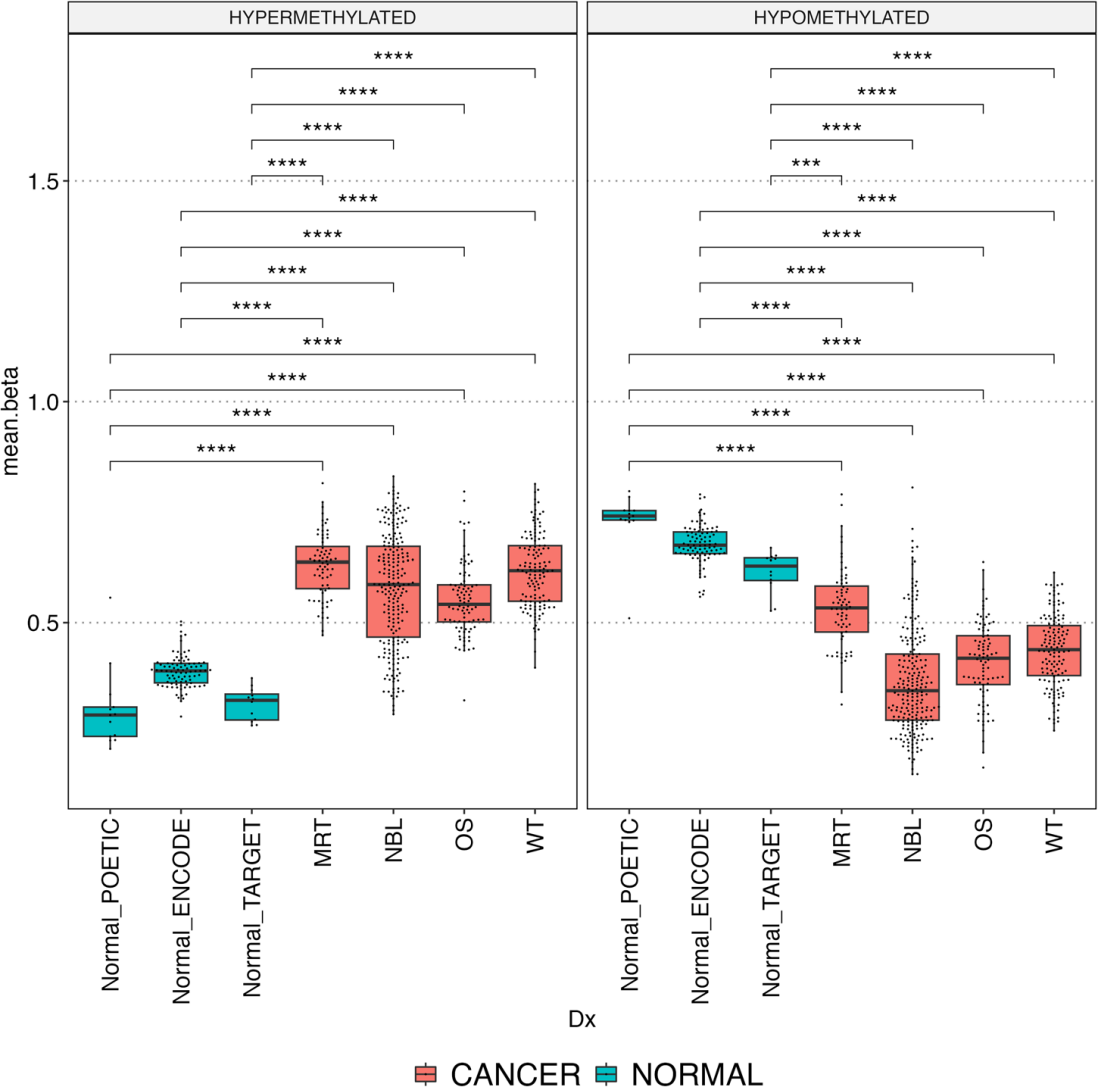
mDMRs in TARGET and ENCODE datasets. Box plots show distribution of per-sample mean beta values across hypermethylated (left) and hypomethylated (right) mDMRs in MRT, NBL OS, and WT tumor samples from the TARGET database. Each point represents one sample, the median beta and interquartile range are indicated by the box plots. Normal tissues from POETIC and normal tissues from ENCODE were used as controls; all comparisons were statistically significant (Wilcox *p*-value ≤ 0.0001) and consistent with the directionality observed in POETIC samples. Significance annotation: ns: *p* > 0.05, *p ≤ 0.05, **p ≤ 0.01, ***p ≤ 0.001, ****p ≤ 0.0001.

Next, a random forest classifier was built using hyper and hypomethylated mDMRs. The cross-validated receiver operating characteristic (ROC) of this classifier model had an area under the curve (AUC) of 0.95, indicating that the mDMRs were capable of differentiating tumor from normal (Fig. 2g). To further validate the mDMRs we analyzed 518 samples (506 cancer, 12 adjacent normal) from the TARGET database (Supplementary Table S6) for Wilms tumor (WT), MRT, osteosarcoma (OS), and NBL samples. We also analyzed 90 normal tissue samples from multiple tissue types from ‘The Encyclopedia of DNA Elements’ (ENCODE) (Supplementary Table S7). Both of these datasets were analyzed using Infinium methylation HM450 and EPIC arrays (Illumina). We were able to assess mean beta value at 344 hypermethylated and 71 hypomethylated mDMRs which overlapped at least 1 probe on the HM450 and EPIC bead chips. We found that methylation patterns of mDMRs in TARGET data resembled POETIC WGBS data, and were hyper or hypomethylated in tumors compared to normal controls (Fig. 3, Wilcoxon *p*-value < 0.001 for all comparisons). Taken together, these results indicate that the mDMRs identified are generalizable across multiple pediatric cancer types.

Hypomethylated mDMRs were located across the genome, with 152 mDMRs within known genes, 30 within 2000bp of a gene transcriptional start site, and 220 in intergenic regions. A majority of these regions were located in open sea (*n* = 328) with 16 regions in CpG islands, 39 in CpG shores, and 18 in CpG shelves. IPA analysis showed genes associated with hypomethylated mDMRs were involved in several immune signaling pathways, including natural killer cell signaling, WNT/β-catenin signaling, and TREM1 signaling (Supplementary Table S8). Hypermethylated mDMRs were more likely to be associated with genes than hypomethylated mDMRs; 316 of 503 regions were within known genes (63% compared to 38% in hypomethylated regions). Most hypermethylated mDMRs were located in CpG islands (*n* = 387) with the remaining mDMRs located in CpG shores (*n* = 6), shelves (*n* = 4), and open sea (*n* = 22). Hypermethylated mDMRs were disproportionately associated with protocadherin genes PCDHGA8, PCDHGA1, PCDHA1, and PCDHA9. IPA found that genes associated with the selected hypermethylated mDMRs were significantly associated with the regulation of epithelial-mesenchymal transition (EMT), NANOG signaling, TGF-signaling, and TREM1 signaling (Supplementary Table S9). Taken together, these results show that the selected mDMRs may represent a pan-pediatric cancer panel, which is associated with cancer-specific pathways.

### mDMRs in pediatric cancer can also be used to detect adult cancers

**Fig. 4:**
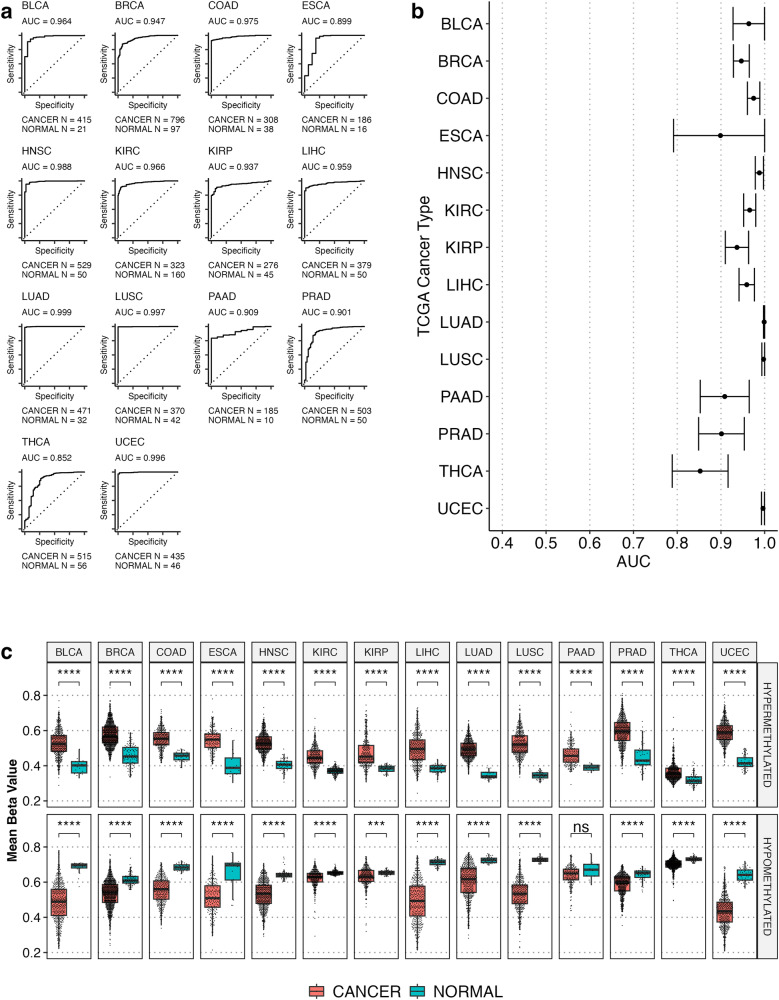
mDMRs detected in multiple adult cancers from TCGA. **a** ROC curves from TCGA 450 K DNA methylation data using a random forest model trained on 422 of the 905 pediatric cancer mDMRs derived by WGBS (subset of 422 regions used due to the limitations of the 450 K array). Plots annotated with TCGA cancer code and AUC. **b** Graphical representation of AUC in A with 95% CI indicated as error bars. See the GDC website for study abbreviation disambiguation (gdc.cancer.gov/resources-tcga-users/tcga-code-tables/tcga-study-abbreviations). **c** Box plots showing per-sample tumor and normal mean beta values across hypermethylated (top) and hypomethylated (bottom) mDMRs in all 14 adult cancer types. All comparisons are statistically significant (Wilcox *p*-value ≤ 0.0001), except PAAD hypomethylated comparison, and consistent with the directionality observed in POETIC samples. Significance annotation: ns: *p* > 0.05, *p ≤ 0.05, **p ≤ 0.01, ***p ≤ 0.001, ****p ≤ 0.0001.

To determine the generalizability of the 905 mDMRs beyond pediatric tumors, we examined a broad set of adult tumor types in TCGA to assess the tumor/normal classifier. Four hundred and twenty two of 905 mDMRs overlapped with at least one probe on the HM450 array used by TCGA (350 hypermethylated mDMRs, 72 hypomethylated mDMRs), and we built a separate random forest classifier based on this reduced set of regions. ROC curves (and AUCs) were calculated for 14 different adult solid tumors (6404 samples – 5691 cancer, 713 adjacent normal tissue, Supplementary Table S10) (Fig. 4a, b). Our model achieved an average AUC of 0.95 indicating that the reduced CpG mDMR set was able to distinguish tumor from normal in samples obtained from TCGA. All but 2 cancer types (THCA – thyroid cancer, and ESCA – esophageal carcinoma) achieved an AUC > 0.9 (Fig. 4c). Performance was consistent across all TCGA tumor stages (SF4-7). This indicates that the panel of CpG mDMRs identified could potentially serve as a pan-cancer methylation early detection panel in both pediatric and adult cancers.

### mDMRs can discriminate CNS tumors from normal tissues

Although the mDMR signature was identified in non-CNS pediatric tumors, we wanted to determine if these regions were also generalizable to pediatric CNS tumors as CNS tumors are the second most common tumor type in children. To do this we downloaded CNS cancer samples profiled by HM450 arrays generated by Capper et al.^10^ hereinafter referred to as ‘DKFZ samples.’ This dataset contains 2682 cancer samples and 119 normal control CNS tissues. The dataset comprises 91 unique methylation classes (Capper et al. Supplementary Table S1) – 82 cancers and 9 normal tissue types. We calculated mean beta values for 422 mDMRs that overlapped with at least one probe on the HM450 array. We found that 44 of 82 tumor types had beta values that were significantly different (Wilcoxon *p*-value < 0.05) between tumor and control tissues with concordant directionality in both hypermethylated and hypomethylated mDMRs (SF8). Next, beta values were fed into the same random forest model used for TCGA samples and ROC curves were calculated for each methylation class using the 119 control CNS tissues as control for each methylation class. The classifier discriminated between tumor and normal with high sensitivity and specificity (AUC > 0.9) in 41/82 CNS tumor types (SF9). Notably, several tumor types in the DKFZ samples were histologically similar to tumor types in the POETIC cohort. For example, three methylation classes – esthesioneuroblastoma (ENB) A, ENB B, and CN NBL – are types of NBL that arise in olfactory nerves and other neural crest cells within the CNS, respectively. The classifier model discriminated tumor from normal tissue in each of these NBL subtypes; CN NBL cases had an AUC = 1, ENB A showed an AUC = 0.97 and ENB B had an AUC = 0.67. Likewise, ATRT DKFZ samples had an AUC = 1. Taken together, these results indicate mDMRs are generalizable to many CNS tumor types and further validated results from POETIC ATRT, NBL and MRT tumor types.

### Correlation of methylation patterns between cell-free DNA and tumor tissue

**Fig. 5:**
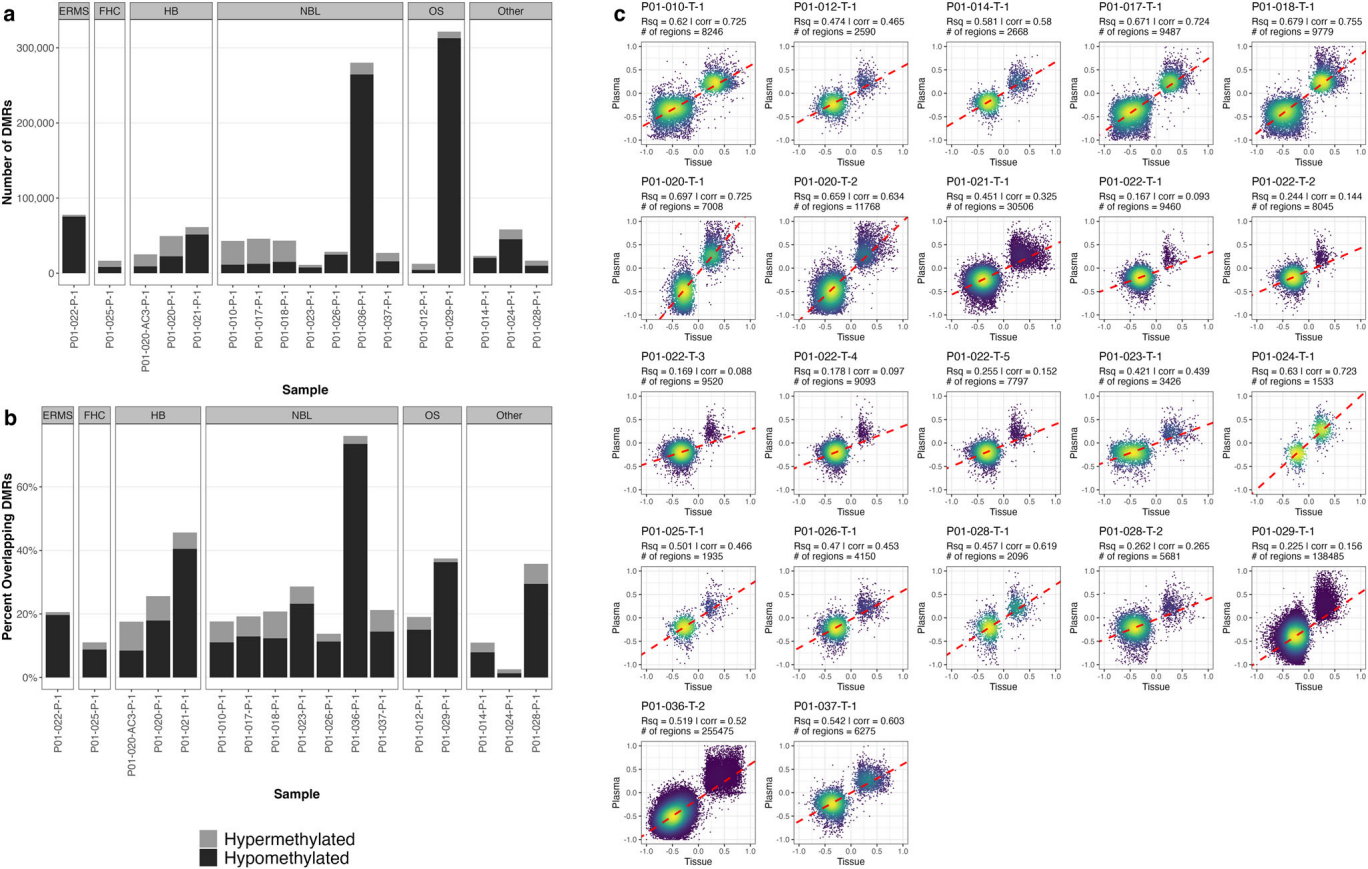
Overlapping DMRs between matching plasma and tissue. **a** Total DMRs called in cell free (cf)DNA. Stacked histograms displaying number of DMRs per sample or **b** percent of DMRs called in cfDNA that overlap with at least one DMR called in patient-matched tumor tissue. Light gray represents the hypermethylated regions and the dark gray represents the hypomethylated regions. **c** Correlation plots of differential methylation of intersecting DMRs between patient matched tissue and cfDNA. Each point represents Δβ in one intersecting region. Plots annotated with R^2^, Spearman correlation, and line of best fit. Color denotes point density (yellow = high, purple = low).

Cell-free DNA methylation is gaining widespread acceptance as an emerging biomarker for liquid biopsies. In the current study, we performed WGBS on 17 individual patient-matched cfDNA samples and 3 healthy young adults to determine the extent to which DNA methylation patterns in genomic DNA (gDNA) can be found in cfDNA (Table 1). CfDNA extracted from 2ml of plasma had an average yield of 20.4 ng (3.12–87.5 ng) with a median concentration of 194 pg/µl (56.8–1590 pg/µl) (SF10). DMRs were called between cfDNA from cancer patients and the three healthy cfDNA samples and filtered in the same way described above for gDNA from tissue. The median number of DMRs was 42,935 per sample (Fig. 5a) (Supplementary Table S11).

We determined the number of overlapping DMRs between cfDNA and gDNA in the patient-matched samples. To count as an overlapping DMR, at least 3 CpGs methylated in the same direction needed to be present in both sample types. The mean percentage of overlapping DMRs between the cfDNA and gDNA (Fig. 5b) was 24.9%^21^. Some notable exceptions include the following samples which had exceptional overlap between cfDNA DMRs and gDNA DMRs: P01-036 (NBL, 76.0%), P0-021 (hepatoblastoma (HB), 45.6%), P01-028 (Teratoma, 35.8%) and P01-029 (OS, 37.4%). The DMRs that overlapped between gDNA and cfDNA were positively correlated (Fig. 5c) with an average R^2^ of 0.45.

### Pan-pediatric mDMRs detectable in cfDNA

**Fig. 6:**
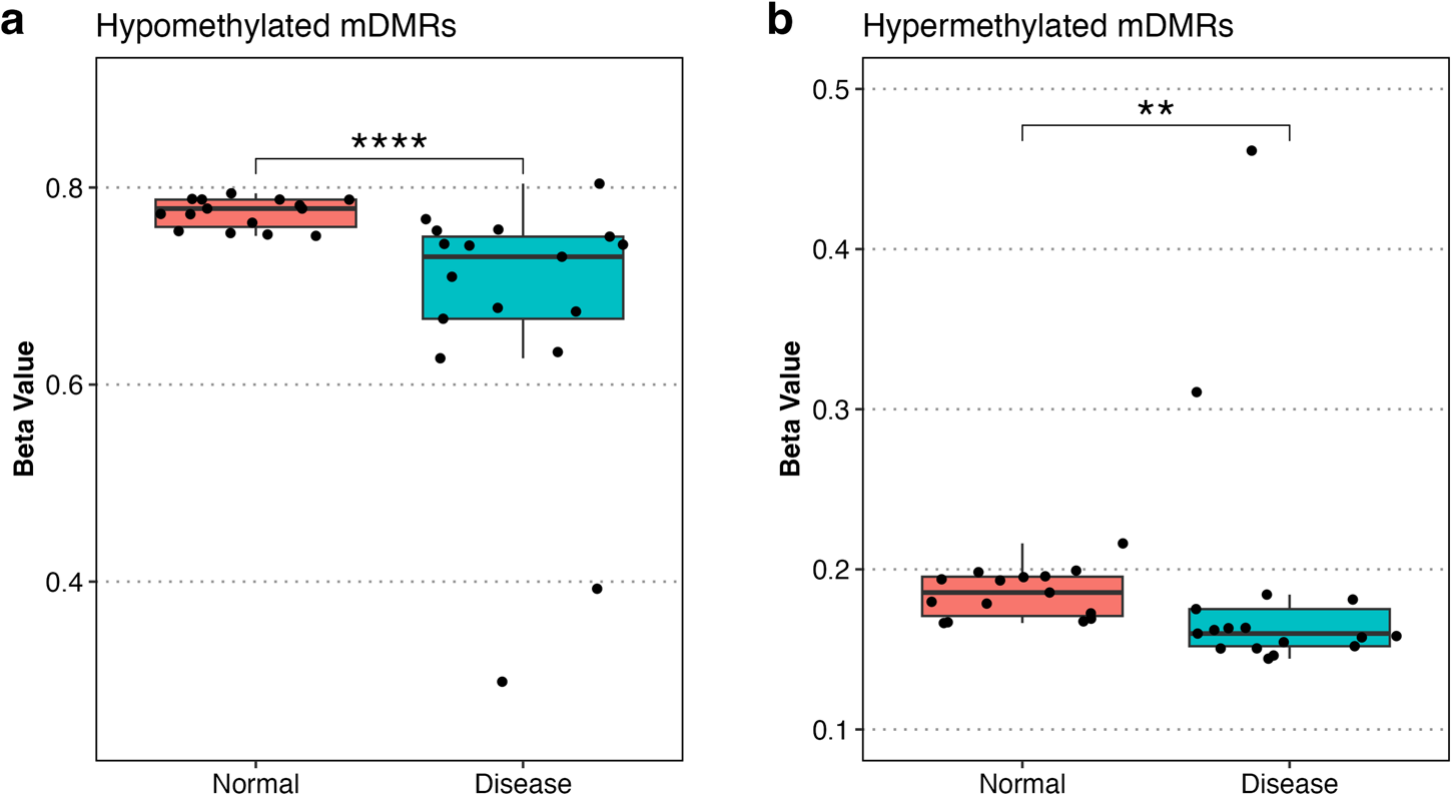
Cell-free DNA methylation analysis of mDMRs in plasma of pediatric cancer samples. **a**, **b** Mean beta from cfDNA across 402 hypomethylated and 503 hypermethylated mDMRs identified in tumor samples. All regions across all healthy and cancer plasma samples are represented.

To determine whether the 905 mDMRs (402 hypomethylated and 503 hypermethylated) across tumor types (Fig. 2) could differentiate between tumor and normal in cfDNA, we performed WGBS in 17 pediatric cancer cases 15 healthy controls (3 from young adults and 12 additional healthy cfDNA samples from adults, Table 1) and calculated mean beta values across all hyper/hypomethylated mDMRs identified in patient-matched tumor tissue, in each WGBS cfDNA sample. The mDMRs which were hypomethylated in tissue were also hypomethylated in cfDNA from cancer patients when compared to cfDNA from healthy samples (normal median meta = 0.779, tumor median beta = 0.730, Wilcoxon *p-value* < 0.0001) (Fig. 6a, Supplementary Fig. SF11). However, mean methylation for hypermethylated regions identified in tissue was also unexpectedly lower in cfDNA cancer samples compared to normal (normal median beta = 0.186, tumor median beta = 0.160, Fig. 6b). For this reason, we focused further validation of mDMRs on hypomethylated regions only and designed a targeted hybridization probe capture assay to 402 hypomethylated mDMRs.

**Fig. 7:**
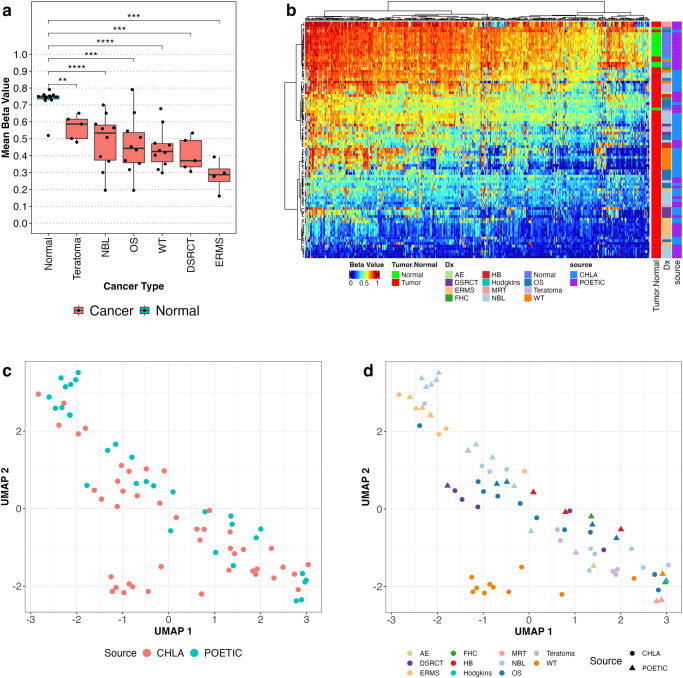
Targeted methylation analysis of hypomethylated mDMRs in an independent cohort of pediatric cancer tissue. **a** Mean beta across 402 hypomethylated mDMRs in 44 additional tissue samples from 6 tumor types acquired from CHLA compared to normal tissue samples. All comparisons are statistically significant (Wilcox rank-sum test, Significance annotation: ns: *p* > 0.05, *p ≤ 0.05, **p ≤ 0.01, ***p ≤ 0.001, ****p ≤ 0.0001). **b** Heatmap of beta values within mDMRs in POETIC and CHLA cohorts. Annotation bars show diagnosis, tumor/normal status, and source for each sample. UMAPs generated using mean beta values across all 402 colored by source (**c**) and cancer type (**d**).

We acquired an additional cohort of 44 pediatric cancer samples from Children’s Hospital Los Angeles (CHLA). cfDNA was not available for these cases. This cohort was comprised of 6 cancer types (NBL, OS, WT, desmoplastic small round cell tumor (DSRCT), embryonal rhabdomyosarcoma (ERMS), and teratoma) (Supplementary Table S12). We found that each of these tumor types was also significantly hypomethylated with respect to normal tissue (Fig. 7a). Hierarchical clustering demonstrated that CHLA samples clustered with POETIC samples and separated from normal tissue samples (Fig. 7b). Furthermore, UMAP analysis did not show a source effect (Fig. 7c) and showed like-tumor types clustering together, regardless of source (Fig. 7d).

### Identification of DNA methylation deserts in neuroblastoma

**Fig. 8:**
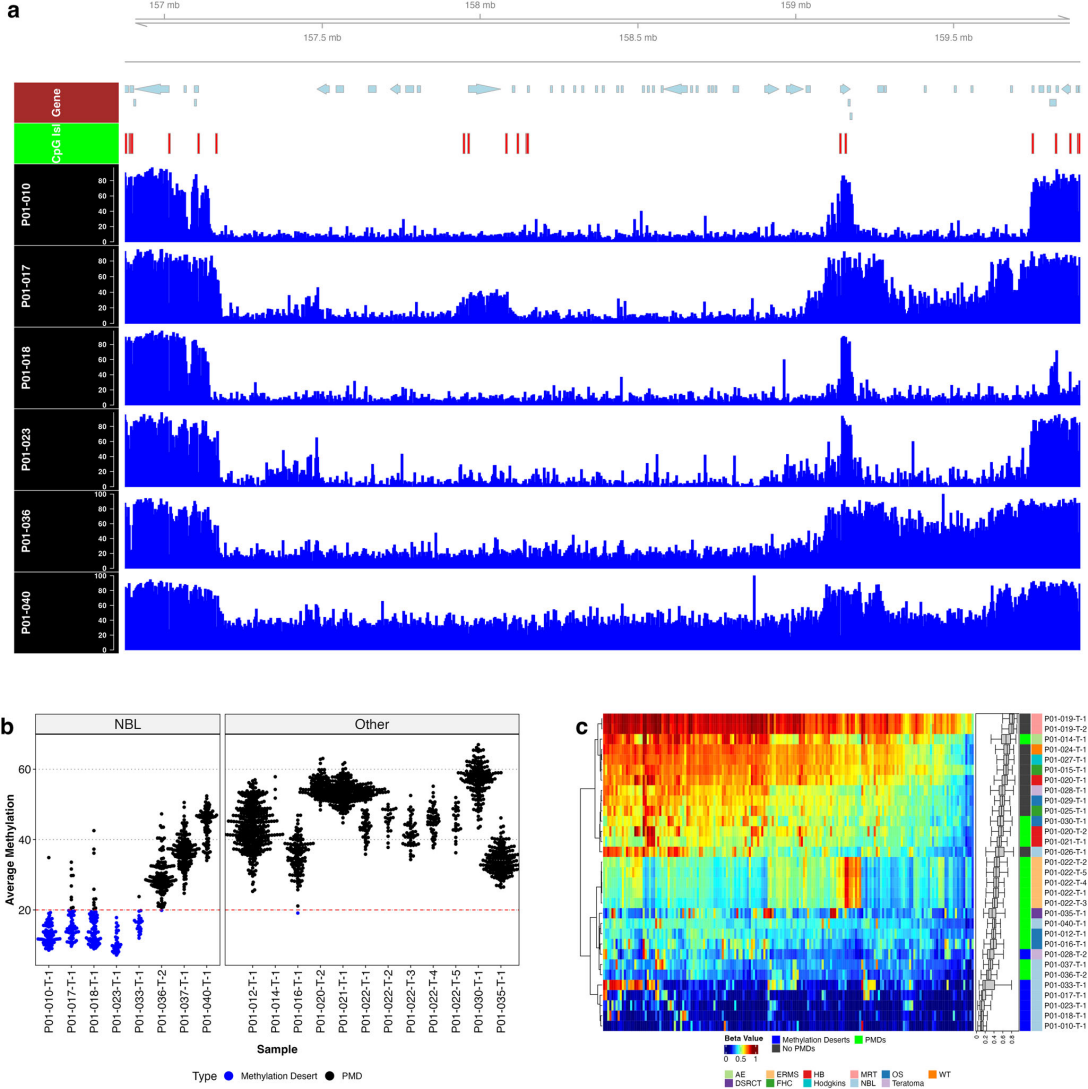
WGBS reveals large regions of eroded DNA methylation termed deserts in neuroblastoma as well as classic partially methylated domains (PMD). **a** DNA methylation deserts in neuroblastoma. Each blue track represents beta values for a different neuroblastoma tumor sample. Beta value from 0 to 100 as indicated on the left of each methylation track. Tracks 1–4 are examples of DNA methylation deserts, and tracks 5–6 are examples of more classic PMD regions. Example region spans 5 Mb. The figure is annotated with chromosome ideogram track (indicating the location of this region on chromosome 1), gene track (light blue arrows denote location and direction of genes) and CpG island track (red blocks). **b** Beeswarm plot of Methpipe PMD analysis. In addition to PMD identification in samples, Methpipe analysis also identified the samples with DNA methylation deserts highlighted in blue. Methpipe calls only regions larger than than 1 Mb in each sample. Y-axis indicates mean beta value in each PMD/methylation desert. Size of each point indicates the width of each region. Plot subdivided by neuroblastoma samples and other tumor types. **c** Heatmap of beta values within PMDs/methylation deserts frequently observed. Row annotation bar 1 denotes whether or not a sample had methylation deserts, PMDs, or neither.

After identifying and validating common focal epigenetic alterations, we turned our focus to large scale genomic and epigenomic alterations such as partially methylated domains (PMDs). PMDs are large regions of hypomethylation spanning several hundred kilobases to several megabases and have been described as a common epigenetic alteration in cancer^22^. PMD-positive samples had an average of 98 PMDs larger than 1 megabase (see Fig. 8a for a PMD example). We also identified multi-kilobase regions that were significantly more hypomethylated, beyond the levels traditionally reported for PMDs, which we referred to as DNA methylation deserts. These deserts were found in 5 of 9 neuroblastomas, but were not identified in any other tumor type. Methylation deserts were characterized by DNA methylation beta value averages less than 0.2, which is significantly lower than that seen in traditional PMDs which typically have a beta value of less than 0.7^22^ (Fig. 8b). PMDs and methylation deserts were also observed in cfDNA in P01-010, P01-029, and P01-036 (SF12).

Interestingly, forkhead box genes FOXA1 and FOXP2 were found within the methylation deserts of the 5 NBL samples. Traditional PMDs were also detected in several other tumors including 3 additional NBL and 11 other samples, including the 5 EMRS samples from one patient, 3 OS, and 2 HB. We also examined copy number variants across POETIC samples to determine if PMDs and methylation deserts were associated with large structural variants. We used permutation testing^23^ to evaluate whether these PMDs and methylation deserts were associated with copy number variants; we did not find a significant association. Further, we plotted the distribution of gDNA copy number within PMDs and methylation deserts and did not observe a shift towards copy number loss or gain within these regions (SF13). We identified 178 regions that contained PMD calls in more than 50% of samples; these regions ranged from 5 kb to 2.1 Mb with an average width of 308 kb and an average of 2699 CpGs per region. Hierarchical clustering of samples based on overall methylation values within these 178 consensus regions separated the 5 NBL samples with deserts from the 14 samples with PMDs and from the remaining samples (with no significant PMDs called) (Fig. 8c).

### Copy number estimation by WGBS in gDNA from tumor tissue

**Fig. 9:**
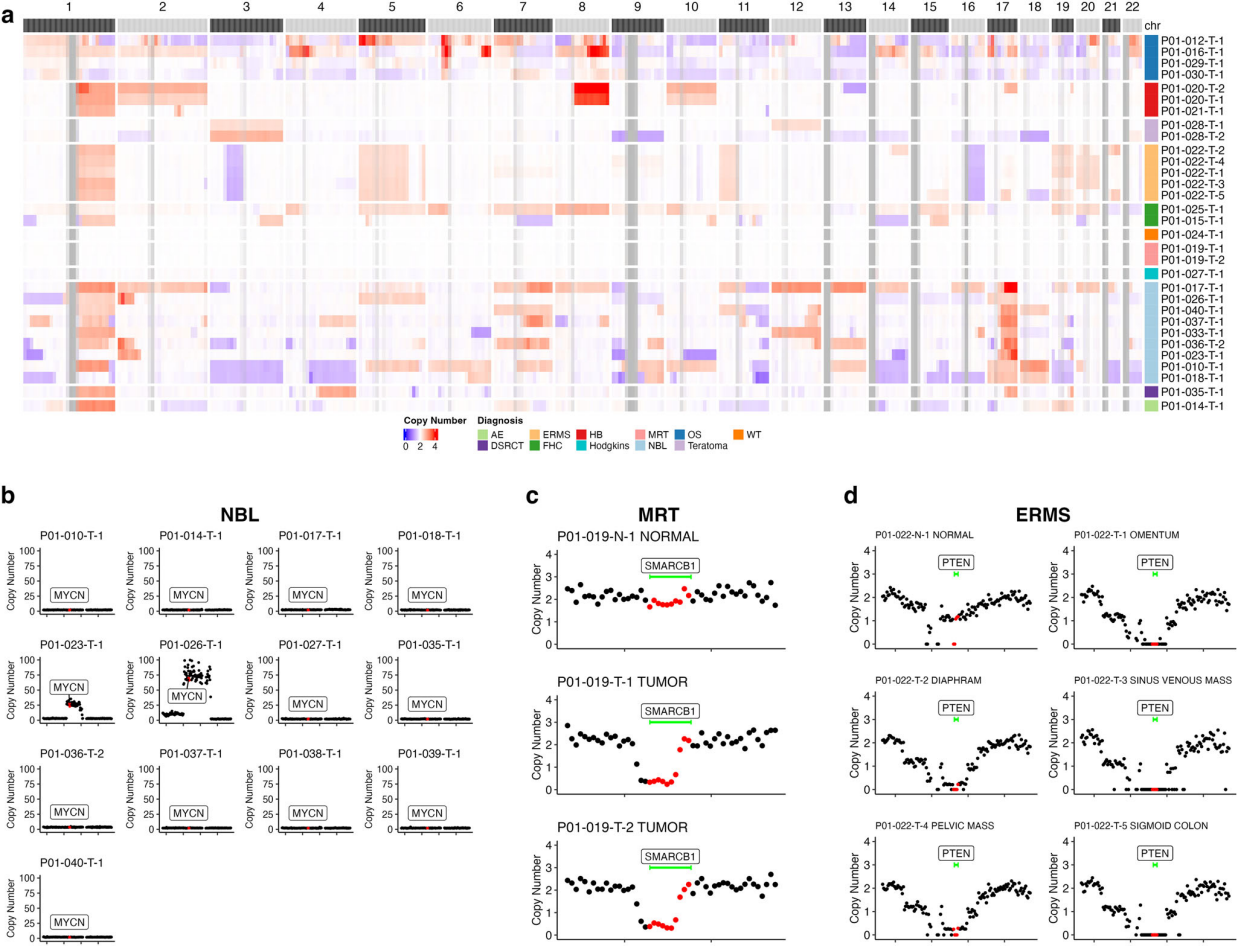
Copy number estimation from WGBS in pediatric cancer tissue. **a** Genome wide copy number evaluation by WGBS across all POETIC tumor samples. Genome-wide chromosome plot (column) of samples grouped by tumor type (rows). Red indicates copy number gain, blue indicates copy number loss, white indicates normal copy number (diploid), and gray indicates that the region was not evaluated. Centromeric regions and p-arms of acrocentric chromosomes were not evaluated and thus appear as gray blocks. Note: copy number scale limited to a maximum of 4 but in some cases the actual copy number may be higher. **b** Copy number of MYCN in neuroblastoma samples. The red points represent the location of the MYCN gene. Each point represents a 5 kb region. **c** SMARCB1 copy number in a malignant rhabdoid tumor. SMARCB1 region indicated by green bar and red points. Top panel is patient matched normal. Middle and bottom panel represent 2 tumors from the same patient. Each point on plot represents a 5 kb region. **d** Loss of PTEN in ERMS. PTEN region indicated by green bar and red points. Top left panel is the patient matched normal sample. Each other panel represents PTEN copy number profiles in tumors from different metastatic sites – indicated in each plot title. Each point on plot represents a 30 kb region.

WGBS is primarily used to measure DNA methylation across the entire genome but can also be used reliably in detecting copy number variations (CNVs)^24,25^. In this study, we conducted copy number analysis from WGBS data, which revealed CNVs in recurrent tumors consistent with known copy number alterations in various primary tumor types studied (Fig. 9a). Specifically, we identified 1q gain – a structural variant associated with poor prognosis^26^ – in 17 of the 31 solid tumor samples (1/1 anaplastic ependymoma (AE), 1/1 DSRCT, 7/10 NBL, 5/5 ERMS, and 3/3 HB). The strong 8q gain in the two hepatoblastoma tumor samples is likewise associated with poor prognosis^27^. 8q was amplified in P01-020 with an average copy number of 3.7 in the first sample collected (P01-020-T-1) and 4.8 in the second sample collected six months later (P01-020-T-2). The copy number profile in these two samples was largely consistent; however, 13q loss and 18q gain were observed at the second time point but not the first. In NBL samples, we observed 1p loss in 4/10 samples, 17q gain in all samples, 11q loss in 6/10 samples, and 3p loss in 5/10 samples. All of these structural variants are relatively common in NBL^28^. In OS samples (P01-012, P01-016, P01-029, and P01-030), copy number changes were widespread and chaotic, a characteristic of OS which often has numerous structural alterations and chromothripsis^29^. The four samples with a hypermethylator phenotype samples had no large chromosomal aberrations: P01-019-T-1 (MRT), P01-019-T-2 (MRT), P01-027-T1 (Hodgkin’s lymphoma), and P01-024-T-1 (WT).

We also detected numerous focal deletions and amplifications that are known to drive the specific tumor types in our study. We were able to observe N-MYC amplification in two of ten NBL samples (Fig. 9b). N-MYC amplifications in NBL occur in 20–25% of NBLs and are associated with poor prognosis^28^. We identified homozygous loss of SMARCB1 in the 2 MRT samples (Fig. 9c), which is a known driver alteration for that tumor type^30^. We observed homozygous loss of MMP11 in MRT. MMP11 CNVs have been reported in NBL and WT in the catalog of somatic mutations in cancer (COSMIC); however, MMP11 CNVs in MRT have not been reported. All samples from P01-022 (ERMS) had PTEN loss, a CNV that has been previously reported and is associated with an aggressive cancer phenotype^31,32^ (Fig. 9d). This homozygous loss in P01-022 also contains ATAD1 which has been associated with cancer cell progression^33^. PTEN loss in this ERMS tumor likely represents a driver tumor event present in all cells.

### Detection of CNVs in cfDNA is less sensitive than cfDNA methylation

**Fig. 10:**
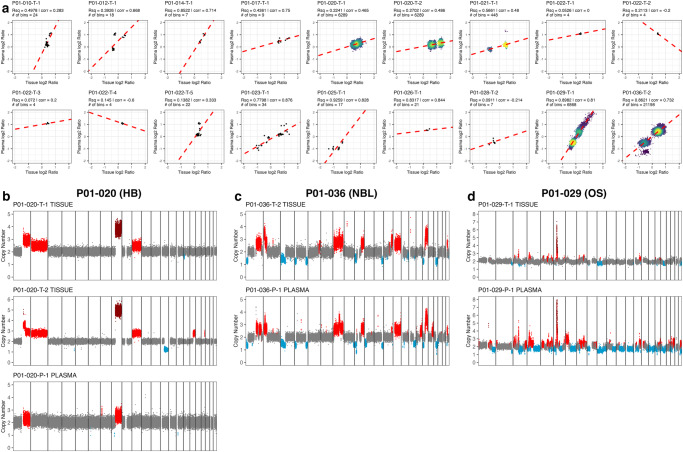
Detection of CNVs in cell-free DNA. **a** Correlation plots of intersecting CNV calls between patient-matched tumor and cfDNA samples. Axes scaled by log2(CGH) ratio. Each point represents a 30 kb bin within overlapping CNV calls between tissue and cfDNA as determined by QDNAseq. Plots annotated with R^2^, Spearman correlation, line of best fit, and number of bins represented in each plot. Genome wide copy number plots for samples with a high number of overlapping regions between tumor and cfDNA: P01-020 (**b**), P01-036 (**c**), P01-029 (**d**). Plasma samples and tumor samples are annotated in the figure. Color indicates the copy number call for discrete regions (dark red = 4 copies gained or greater, red = 3, gray = 2, light blue = 1). Vertical lines delineate individual chromosomes.

Next, we used WGBS data to analyze CNVs in cfDNA. Unlike many known CNVs found in tissue gDNA, CNVs were largely not observable in cfDNA (Fig. 10a). Five samples from three patients represented notable exceptions (Fig. 10b–d), where CNV calls were broadly consistent with CNV detection from tissue gDNA. In one such example (P01-020), the 1q and 8q gains observed in 2 tissue samples were observed in cfDNA (Fig. 10b). Blood from this patient was taken at the time the first tumor sample (P01-020-T1) was collected and both samples had a similar CNV profile. The second tumor sample (P01-020-T2) was collected 9 months after the first sample and showed a 13q loss not seen in the first sample (T1) or its cfDNA. In P01-036 (NBL) the cfDNA CNV calls also closely matched tumor sample CNV data, with gains on 1q, 2p, 7, 9q, 12q, 13q, 17q and losses on 1p, 3p, 4q/p, 11q, 17q, and 19q (Fig. 10c). Interestingly the cfDNA was able to resolve some CNVs not seen in the tissue, namely losses in 10p and 15q. This was also true for P01-029 (Fig. 10d) where there were CNVs in plasma not seen in gDNA. In addition, we were able to identify focal N-MYC amplification in P01-026 but not in P01-023 as previously identified in gDNA (SF14). The reasons why CNV calls in cfDNA were concordant with gDNA in only a few samples are unknown, but appear to be unrelated to tumor cfDNA yields (SF10). Overall, this analysis revealed that detection of tissue-associated DNA methylation in plasma cfDNA is more sensitive than detecting tissue-associated CNV alterations in plasma cfDNA.

## Discussion

In this study we set out to determine the set of DMRs common to multiple pediatric solid tumors. To do this we performed WGBS on 31 tumor and 13 normal tissue samples from recurrent pediatric non-CNS solid cancers representing 11 different tumor types. We further validated DNA methylation findings in an additional 506 pediatric cancer samples from TARGET, 5691 adult cancer samples from TCGA, and 2682 CNS cancer samples generated by Capper et al. along with an additional 44 pediatric cancer tissue samples examined through a targeted hybridization probe capture assay. Additionally, we examined corresponding cfDNA methylation from 17 patient-matched plasma samples. Previous studies have identified DNA methylation changes in many of the pediatric cancers evaluated in this study, including NBL^34^, OS^35,36^, WT^37^, AE^38^, HB^39^, ERMS^39^, and fibrolamellar hepatocellular carcinoma^40^, however methylation analysis on recurrent pediatric cancer cases has been limited^41–43^. Novel to our study is the finding that in addition to tumor-specific changes, DNA methylation patterns were also shared across the myriad of tumor types analyzed regardless of whether a tumor was from a primary or recurrent tumor. Previous work by Yang et al.^44^ demonstrated that there are DNA methylation changes common to multiple adult cancer types, which are frequently associated with tumor suppressor genes, and often associated with survival. However, similar findings in pediatric cancers have been scarce. Given the rarity of many pediatric cancers, identifying DNA methylation patterns across multiple pediatric cancer types might have great value for developing biomarker approaches for early detection and/or disease recurrence through MRD detection.

To find DNA methylation commonalities between cancer types, we identified a minimal region of differential methylation common to multiple samples across cancer types which we termed minimally differentially methylated regions – mDMRs. These mDMRs were significantly differentially methylated in multiple cancer types, including very rare tumors like DSRCT, when compared to normal tissue. We also evaluated 506 pediatric cancer samples from the TARGET database, where we detected methylation alterations in the mDMR regions consistent with those observed in POETIC samples, indicating this set of features is generalizable. Furthermore, since this mDMR panel is derived from recurrent patients, it may reflect a method to conduct recurrence monitoring via MRD detection in pediatric cancer. Additionally, the mDMRs also demonstrated high sensitivity and specificity when tested in adult cancer samples representing 14 cancer types from TCGA with excellent sensitivity and specificity for stage 1 cancers, suggesting that these mDMRs could also serve as a pan-cancer early detection marker in adult cancers. Finally, we found mDMRs discriminated tumor from normal tissue in several CNS tumor types in both adults and children. Another noteworthy finding of this study was that methylation profiles of recurrent POETIC samples largely resemble those of primary tumors sourced from TARGET. Previous work has shown that primary and recurrent tumors have similar methylation profiles^45^, however, this has not been previously reported in pediatric cancer.

Among the tumor-specific DNA methylation changes of interest was the identification of DNA methylation deserts in a subset of NBL cases, where large regions of DNA methylation are essentially eroded. The functional significance of these DNA methylation deserts is unknown but they are distinct from previously reported PMDs by having significantly lower levels of DNA methylation. Previous work by Brinkman et al. found that PMDs are associated with CpG island methylation in breast cancer^46^; PMDs have also been associated with lamina-associated domains (LADs)^47^. Increased expression of FOXA1, one of the few genes found in DNA methylation deserts, has been previously linked to late recurrence^48^. Further research is needed to explore possible mechanisms of these desert regions in NBL. Another tumor-specific methylation change we observed was strong global hypermethylation in MRT – an atypical cancer phenotype in both pediatric and adult tumors. Hypermethylation in cancer typically occurs in the promoter regions of tumor suppressor genes^49–53^ and in CpG islands, where it is associated with poor prognosis in many adult and pediatric cancers and can be associated with CIMP^54–57^. However, global hypermethylation is rarely reported in cancer. While MRT has been previously shown to have focal hypermethylation^58^, the global hypermethylator phenotype we observed has not been previously described. Previous work by Kenny et al. found that SMARCB1 restoration in MRT cell lines resulted in widespread chromatin activation^59^. Conversely this hypermethylator phenotype could be linked to genome-wide chromatin inactivation but future studies would be needed to evaluate if this is true.

Unlike adult cancers, most cases of pediatric cancer can be traced to a single genetic driver, and these genetic alterations tend to be highly tumor-specific. For example, the loss of SMARCB1 specifically leads to the development of MRT^30^. Specific gene fusions cause alveolar rhabdomyosarcoma (PAX3/7-FOXO1)^60^, Ewing’s sarcoma (EWS-FLI1)^61^, and CML (BCR-ABL1)^62^. Mutations in certain genes such as RB are specific to retinoblastoma and OS, while mutations in TP53 are extremely common in multiple pediatric cancers. WGBS is the gold standard assay for methylation evaluation as it evaluates every CpG in the genome but we also used it for copy number estimation – maximizing data generation from each sample and enabling multi-omic analysis from the same sample and aliquot, which also minimizes sampling bias and reduces cost. This is particularly useful when dealing with rare tumor types or sample types with very little available DNA (such as cfDNA). CNV analysis from WGBS identified both large-scale alterations and focal gains/losses such as MYCN gain in NBL, SMARCB1 loss in MRT, and PTEN loss in ERMS.

A key component of this study was evaluating the degree to which genomic alterations in solid tumors were reflected in cfDNA, as cfDNA analysis is rapidly becoming a major tool for non-invasive screening, diagnosis, treatment, and monitoring of human tumors. Previous work in renal cell carcinoma by Lasseter et al.^63^ found that cfDNA methylation (using MeDIP-seq) was far more sensitive and specific than cfDNA SNV markers. In the current study, we found that detecting tumor tissue-derived DNA methylation using WGBS was superior to detecting CNVs in cfDNA. In this study, only 3/17 cfDNA samples reflected the copy number profile of the tumor, whereas the remaining samples lacked much of the signal found in tissue. By contrast, we found cfDNA methylation to resemble tissue DNA methylation more robustly, where on average approximately 25% of DMRs were common to tissue and plasma in each sample. We also found that hypermethylated regions were less likely to be retained in plasma and were often found to be hypomethylated. While the reasons for this are unclear, the implications are significant as it would have implications for biomarker development strategies.

The data presented in this manuscript are novel with respect to identifying a pan-pediatric cancer panel with potentially broader utility to adult tumors and which can be further developed for early detection or MRD biomarkers. Still, our study has some limitations. First, the number of cases for some tumor types (MRT, AE, immature teratoma) was small and we were unable to further validate mDMRs in external datasets or obtain additional tissue samples for these tumor types. While future validation is warranted, the point of our study was intended to lift these otherwise very rare tumor types. Second, additional work is required to further validate mDMRs in cfDNA and to determine if these mDMRs can be used for early detection or MRD monitoring.

In summary, we have performed a comprehensive analysis of multiple pediatric cancers using WGBS. We identified a pan-cancer methylation panel, detectable in cfDNA, common to multiple pediatric cancer types, including extremely rare neoplasms such as DSRCT and MRT. This panel was also generalizable to pediatric CNS tumors and 5691 adult tumors. We also used WGBS to detect CNVs, in order to directly compare CNV and methylation detection from the same sample and aliquot. Lastly, we found that DNA methylation was superior to CNV at detecting tumor-specific signal in cfDNA. The pan-cancer cfDNA methylation panel in this study has potential utility in MRD monitoring and early detection and warrants further investigation in both pediatric and adult cancer.

## Methods

### Sample collection

Samples were obtained under written informed parental consent from the Pediatric Oncology Experimental Therapeutics Investigators’ Consortium (POETIC) at Memorial Sloan Kettering Cancer Center (New York, USA) from patients with a wide range of recurrent pediatric cancers (Table 1). Tissue samples were flash-frozen after resection. Peripheral blood samples were drawn pre-operatively in EDTA purple-top tubes and the plasma was harvested.

All participants in the study were patients with recurrent pediatric cancer. In total, 44 tissue samples were collected including 31 tumor samples and 13 matching adjacent normal samples from 24 patients. All tissue samples were collected during surgical resection of the recurrent tumor. 17 patient-matched plasma samples were collected, with 3 additional plasma samples from young healthy individuals purchased from Conversant Bio (Table 1). Twelve additional cfDNA healthy control samples from adults were included for model building in cfDNA, detailed below (Conversant Bio) (Table 1). Patients ranged in age from 18 months to 35 years. In total, 11 different cancer types were included in the study: neuroblastoma (NBL, *n* = 9), osteosarcoma (OS, *n* = 4; 3 from pulmonary metastasis, 1 from retroperitoneal metastasis), fibrolamellar hepatocellular carcinoma (FHC, *n* = 2), hepatoblastoma (HB, *n* = 2; both resected from the liver, with one additional sample from a pulmonary metastasis), anaplastic ependymoma (*n* = 1), desmoplastic small round cell tumor (*n* = 1; from a gastric lesion), malignant rhabdoid tumor (MRT, *n* = 1; 2 samples from an abdominal lesion), Hodgkin’s lymphoma (*n* = 1; from a lung biopsy), embryonal rhabdomyosarcoma (ERMS, *n* = 1; with 5 samples from omentum, diaphragm, sinus venous, pelvic, and sigmoid colon lesions), immature teratoma (*n* = 1; with 2 samples from cul de sac and ileum lesions), and Wilms’ tumor (*n* = 1).

We also obtained a separate cohort of 44 pediatric cancer tissue samples for validation from the Center for Pathology Research Services at Children’s Hospital Los Angeles (CHLA). These samples included 6 cancer types (NBL *N* = 10, OS *N* = 10, WT *N* = 10, DSRCT *N* = 5, Teratoma *N* = 5, ERMS *N* = 4) and were collected under written informed consent and preserved in OCT compound (Supplementary Table S12). In addition, we included 68 MRT, 221 NBL, 86 OS, 131 WT, and 12 adjacent normal tissue samples from TARGET (total = 518), 90 normal tissue samples from ENCODE, and 5691 adult cancer samples from TCGA from 14 tumor types along with 713 adjacent normal samples. We also accessed 2682 CNS cancer samples and 119 normal control CNS tissues generated by Capper et al.^10^ (GEO accession GSE90496). All four of these cohorts were analyzed on the HM450 or EPIC arrays (Illumina) (Supplementary Tables S6, S7 and S10).

### Sample extraction and library preparation for whole genome bisulfite sequencing

Genomic (g)DNA was extracted from flash-frozen normal or tumor tissue using an AllPrep DNA/RNA Mini kit (Qiagen) according to manufacturer’s recommendations. Briefly, tissues were homogenized using a Bullet Blender homogenizer (Next Advance) for 5 min at full speed with a mixture of 0.9–2.0 mm RNase-free stainless-steel beads. Homogenates were passed through the QIAshredder (Qiagen) to remove any remaining particulate matter. Plasma was harvested prior to surgery at Memorial Sloan Kettering Cancer Center in EDTA (purple-top) tubes and shipped on dry ice to the Salhia Lab at the University of Southern California. Cell-free (cf)DNA was extracted from the plasma using the MagMax Cell-Free DNA Isolation Kit (Thermo Fisher) according to the manufacturer’s recommendations.

Quantity and purity of the isolated gDNA was determined by TapeStation using gDNA tapes (Agilent). cfDNA was quantitated using the TapeStation High Sensitivity D1000 assay according to the manufacturer’s protocol. Extracted cfDNA was used for WGBS as previously described^64^ at the Keck Genomics Platform, University of Southern California. Briefly, directional bisulfite-converted libraries for paired-end sequencing were prepared using the Ovation Ultralow Methyl-Seq Library System (NuGen), using the manufacturer’s suggested protocol. Bisulfite conversion was performed using the EpiTect Fast DNA Bisulfite Kit (Qiagen). Post-library QC was performed on the 4200 Tapestation using High Sensitivity D1000 ScreenTapes (Agilent). Paired-end sequencing was performed on the Illumina NovaSeq 6000 platform using the S2 or S4 flow cell for a total read length of 2 × 150 bp. Genomic DNA from tissue samples was sequenced at Macrogen Clinical Laboratory (Rockville, MD). Libraries were constructed using Illumina’s TruSeq DNA Methylation Kit. Paired-end sequencing was performed on a HiSeq X (Illumina) sequencer with a total read length of 2 × 150 bp. A 20% PhiX control spike-in was used to ensure diverse cluster generation. Read pairs were processed through our alignment and methylation calling pipeline which uses Brabham Bioinformatics’ Bismark alignment software^65^ for read mapping and methylation evaluation. All reads were mapped to hg19.

### DMR calling

Differentially methylated regions (DMRs) were evaluated using the DMR caller Metilene^66^. Tumor samples were compared with their patient-matched adjacent normal sample where possible. For tumor samples without a matched normal, a pool of normal samples from other patients with the same diagnosis was used. For unique cancer types with no matched normal sample, a pool of all normal samples was used. DMRs were filtered to include those with a Mann-Whitney *p*-value < 0.05 and an |Δβ| > 0.15. Regions from X and Y chromosome were removed. DMRs were annotated with the name of the nearest gene. Overlapping DMRs were defined as regions in which DMRs for two or more samples shared at least 3 CpGs, with the same directionality (hyper- vs. hypo-methylated).

### Partially methylated domain/hypomethylated domain analysis

Partially methylated domains (PMDs) are hypomethylated domains with an intermediate level of methylation spanning several kilobases to a few megabases. PMDs were called using MethPipe^67^, based on methylation and coverage information from Bismark. We modified methPipe’s code to generate additional metrics, namely mean methylation across each PMD and the standard deviation of beta values within each PMD call. Analysis and plotting of PMD regions were performed in R.

### CpG minimally differentially methylated regions

We identified a consensus DMR set with substantial enrichment of hypomethylated and hypermethylated DMRs across all samples. Each CpG location was scored by the number of samples with a DMR overlapping this locus (separately for hyper- and hyper-methylated DMRs). These CpGs were filtered to include those with a count of 22 or greater (70% of samples had a DMR call that overlapped that locus), and filtered CpGs within 500 bases of each other were combined to form separate regions, termed minimally differentially methylated regions (mDMRs).

### Copy number analysis

Copy number variants (CNVs) were called on the WGBS data using the R package QDNAseq^68^, which uses read counts within fixed sized bins; we selected a 30 kb bin size for evaluation of genome wide copy number variants, and a 1 kb, 5 kb, or 10 kb bin size for focal amplifications such as MYCN gain in neuroblastoma.

### Ingenuity pathway analysis

In order to investigate biological pathways associated with DMRs we used Ingenuity Pathway Analysis (IPA, Qiagen). DMRs were annotated with the nearest gene and distance to the nearest gene. The resulting gene list was used as IPA input. We ran pathway enrichment analysis using default IPA settings.

### Machine learning classifiers

A mean beta value matrix was used to construct a random forest model using ranger. We used repeated (*n* = 10) 3-fold cross validation to estimate the performance of all models. For the classification of TCGA solid tumors, a single model constructed from beta value of all pediatric solid tissues was used.

### Hybridization probe capture

Hybridization probe capture, assay design, sequencing, and bioinformatic analysis were performed as previously described^69,70^.

### Ethics

Patient tissue and blood samples and associated clinical and demographic data (eg. age, diagnosis) were collected under written informed consent at Memorial Sloan Kettering Cancer Center (on behalf of the Pediatric Oncology Experimental Therapeutics Investigators’ Consortium - POETIC) and Children’s Hospital Los Angeles. Samples were deidentified and shipped to USC under an approved IRB protocol (IRB#: HS-16-00540, USC Institutional Review Board) for tissue and blood collection for the study as well as under a material transfer agreement and in compliance with the regulations laid forth in the Declaration of Helsinki.

All patient samples were deidentified and assigned a unique sample ID before sending to USC. Relevant local and federal regulations with regards to information privacy, including the Healthcare Insurance Portability and Accountability Act (HIPAA) and IRB mandated patient privacy protections, were adhered to. Only those study personnel involved with clinical annotation of study data had access to identifiable information linking patients to their samples.

All study personnel involved were required to have annual certification/re-certification of human subjects training (CITI or equivalent) prior to any participation in clinical research activities.

### Reporting summary

Further information on research design is available in the Nature Research Reporting Summary linked to this article.

### Supplementary information

Supplemental Information

REPORTING SUMMARY

## Data Availability

The IRB protocol for this study does not permit data to be submitted to an online database nor distributed via a data use agreement.

## Code availability

Open-source bioinformatic tools used for analysis are cited in the methods section. Requests for R code used for downstream analysis should be directed to and will be fulfilled by the Lead Contact, Bodour Salhia (salhia@usc.edu).
