# Supplementary material for: A comprehensive analysis of minimally differentially methylated regions common to pediatric and adult solid tumors
Source: NPJ Precis Oncol. 2024 Jun 1;8:125. doi: 10.1038/s41698-024-00590-1 (PMC11144230; doi:10.1038/s41698-024-00590-1)
Supplement: Supplementary file 1 — Supplemental Information [file 41698_2024_590_MOESM1_ESM.pdf]

A Comprehensive Analysis of Minimally Differentially Methylated Regions Common to Pediatric and Adult Solid Tumors: Supplementary Information

April 2, 2024

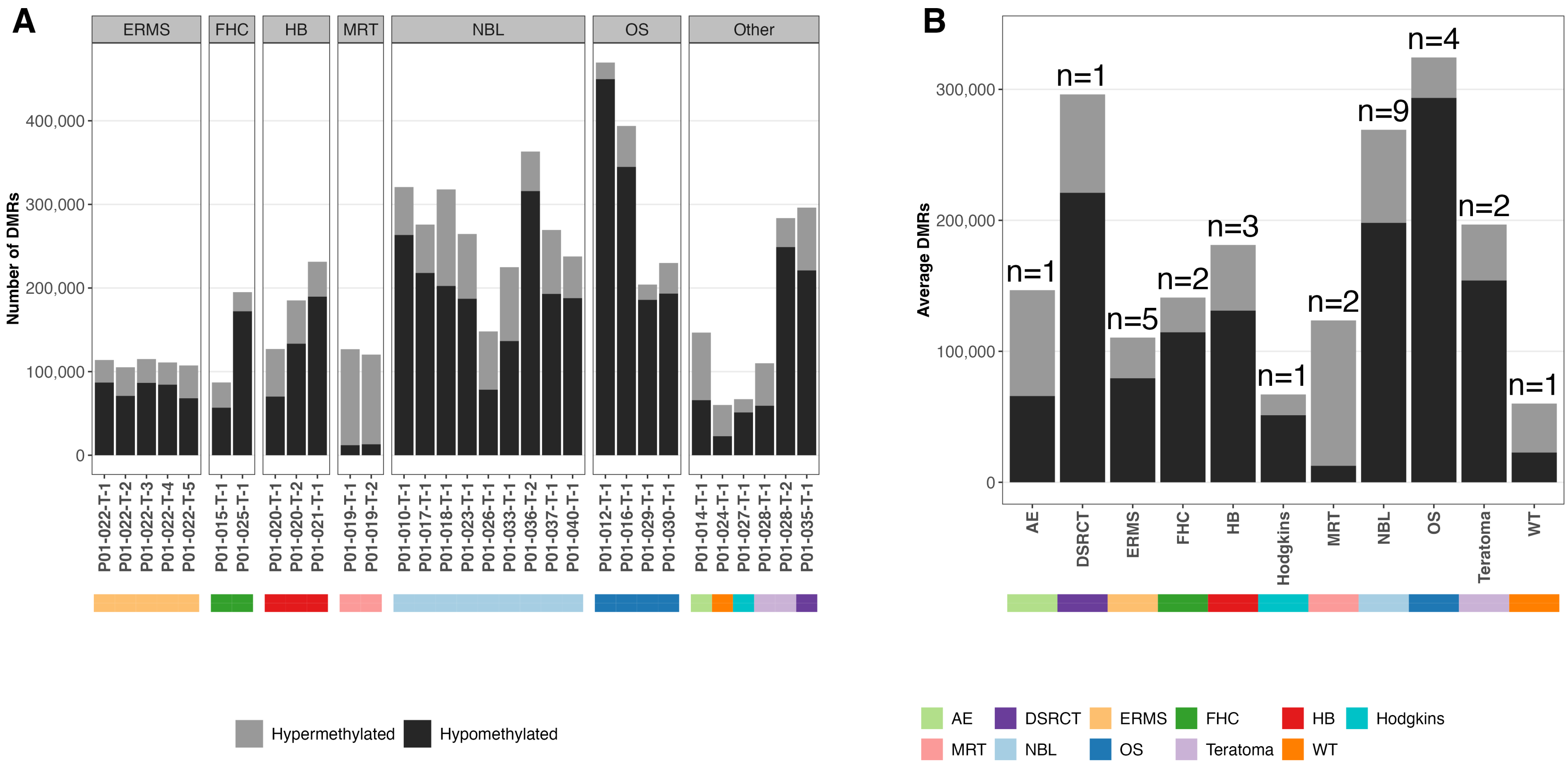

Supplemental Figure 1: Number of DMRs called in WGBS by sample and cancer type.
(A) Number of DMRs per sample. Stacked histograms displaying number of DMRs per sample. Light grey represents the hypermethylated regions and the dark grey represents the hypomethylated regions. Chart is subdivided by tumor type: Embryonal rhabdomyosarcoma (ERMS), neuroblastoma (NBL) and osteosarcoma (OS), hepatoblastoma (HB), malignant rhabdoid tumor (MRT), and fibrolamellar hepatocellular carcinoma (FHC); all cancer types were grouped as 'Other'. **(B)** Stacked Histogram displaying average number of DMRs per cancer type, where n denotes number of samples per tumor type.

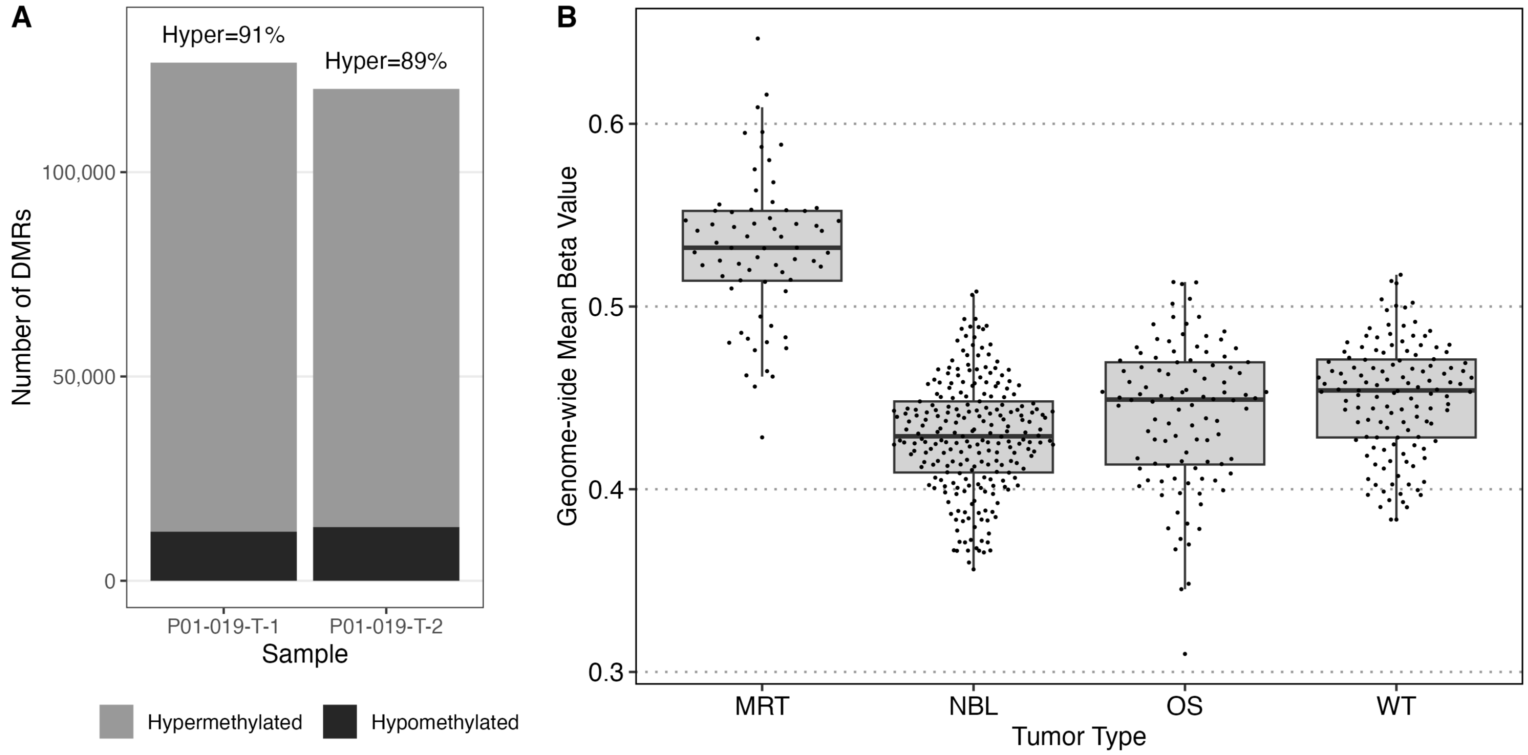

Supplemental Figure 2: MRT methylation profiles.
(A) Number of DMRs called in P01-019 (MRT). Shade delineates hypermethylated regions (light grey) from hypomethylated regions (dark grey). Each sample annotated with percent of DMR calls that are hypermethylated. **(B)** Genome-wide beta value of TARGET samples shows MRT hypermethylation.

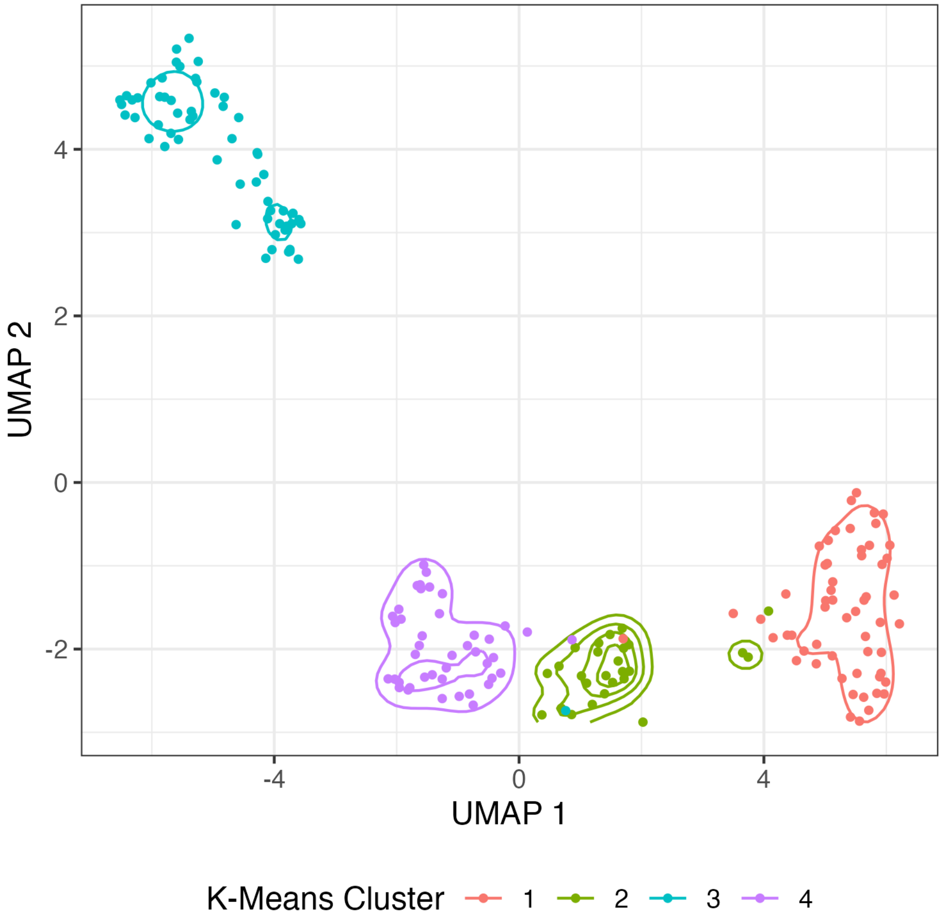

Supplemental Figure 3: UMAP of DMRs identified in Figure 1c by k-means cluster.
Each point represents an individual DMR as detailed in Figure 1c. Clusters on column annotation of Figure 1c are 'K-Means Cluster'.

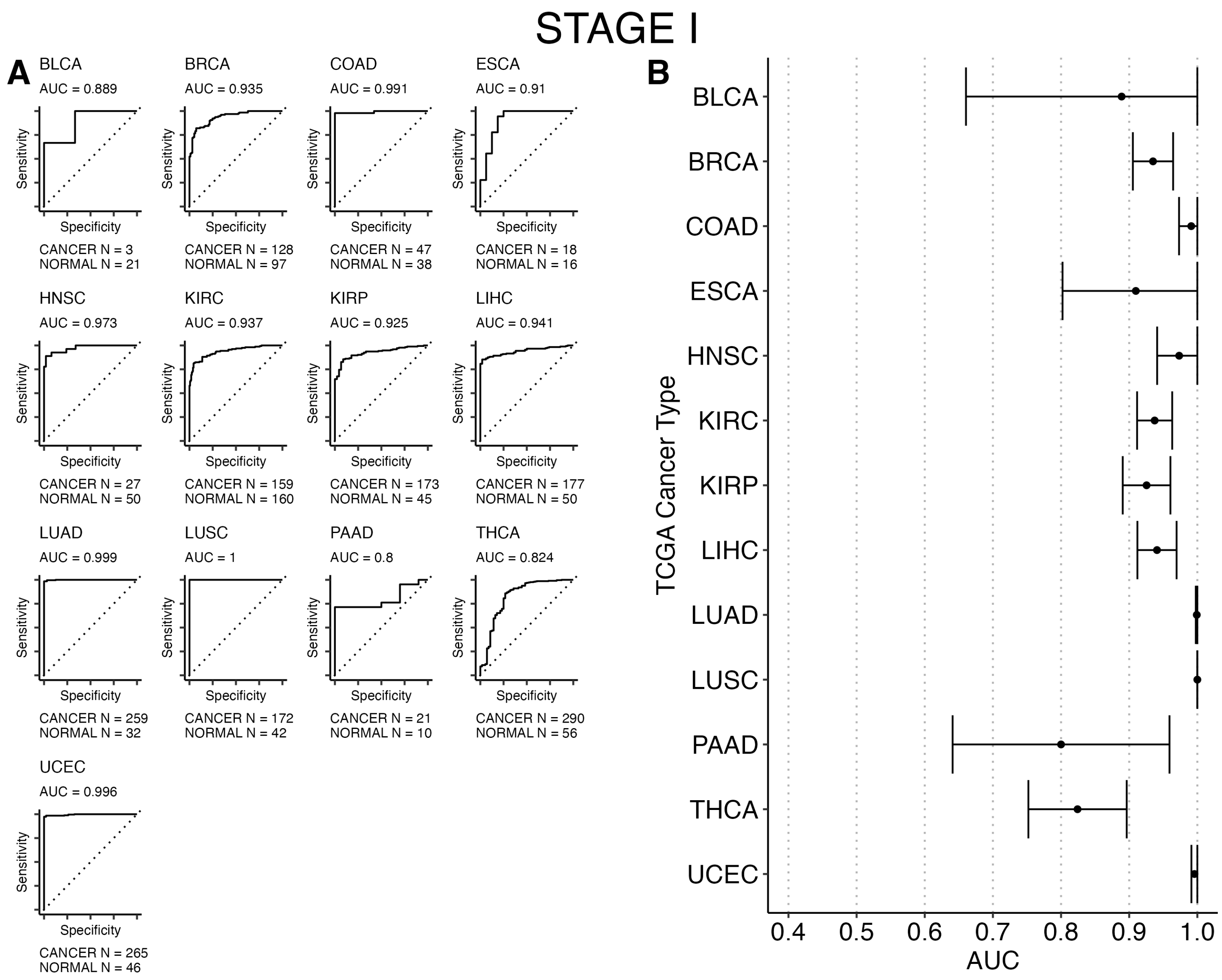

Supplemental Figure 4: mDMRs detected in multiple stage I adult cancers from TCGA
(A) ROC curves from TCGA 450K DNA methylation data using a random forest model trained 422 of the 905 pediatric cancer mDMRs derived by WGBS (subset of 422 regions used due to the limitations of the 450K array). Plots annotated with TCGA cancer code and AUC. **(B)** Graphical representation of AUC in A with 95% CI indicated as error bars. See the GDC website for study abbreviation disambiguation (gdc.cancer.gov/resources-tcga-users/tcga-code-tables/tcga-study-abbreviations).

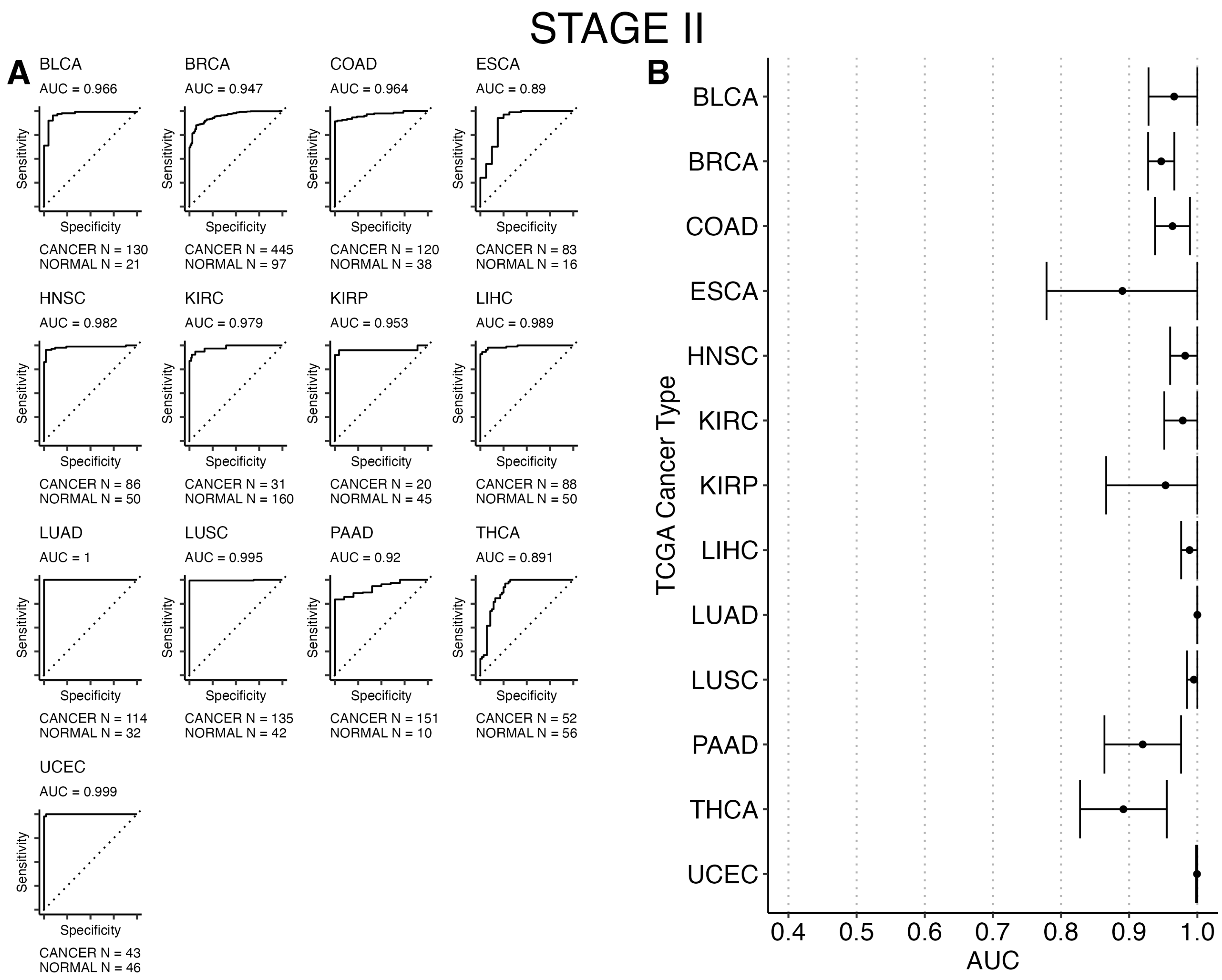

Supplemental Figure 5: mDMRs detected in multiple stage II adult cancers from TCGA
(A) ROC curves from TCGA 450K DNA methylation data using a random forest model trained 422 of the 905 pediatric cancer mDMRs derived by WGBS (subset of 422 regions used due to the limitations of the 450K array). Plots annotated with TCGA cancer code and AUC. **(B)** Graphical representation of AUC in A with 95% CI indicated as error bars. See the GDC website for study abbreviation disambiguation (gdc.cancer.gov/resources-tcga-users/tcga-code-tables/tcga-study-abbreviations).

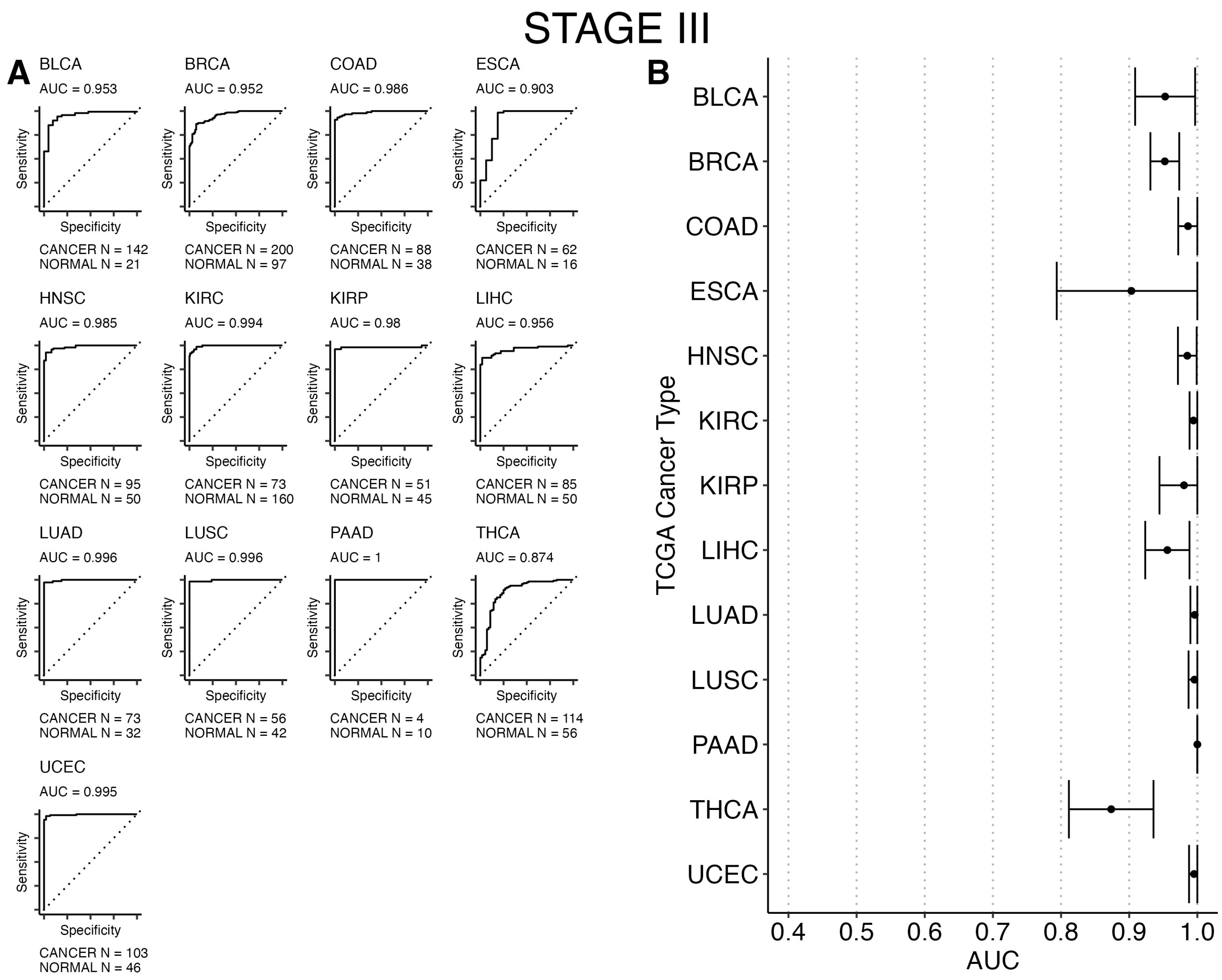

Supplemental Figure 6: mDMRs detected in multiple stage III adult cancers from TCGA
(A) ROC curves from TCGA 450K DNA methylation data using a random forest model trained 422 of the 905 pediatric cancer mDMRs derived by WGBS (subset of 422 regions used due to the limitations of the 450K array). Plots annotated with TCGA cancer code and AUC. **(B)** Graphical representation of AUC in A with 95% CI indicated as error bars. See the GDC website for study abbreviation disambiguation (gdc.cancer.gov/resources-tcga-users/tcga-code-tables/tcga-study-abbreviations).

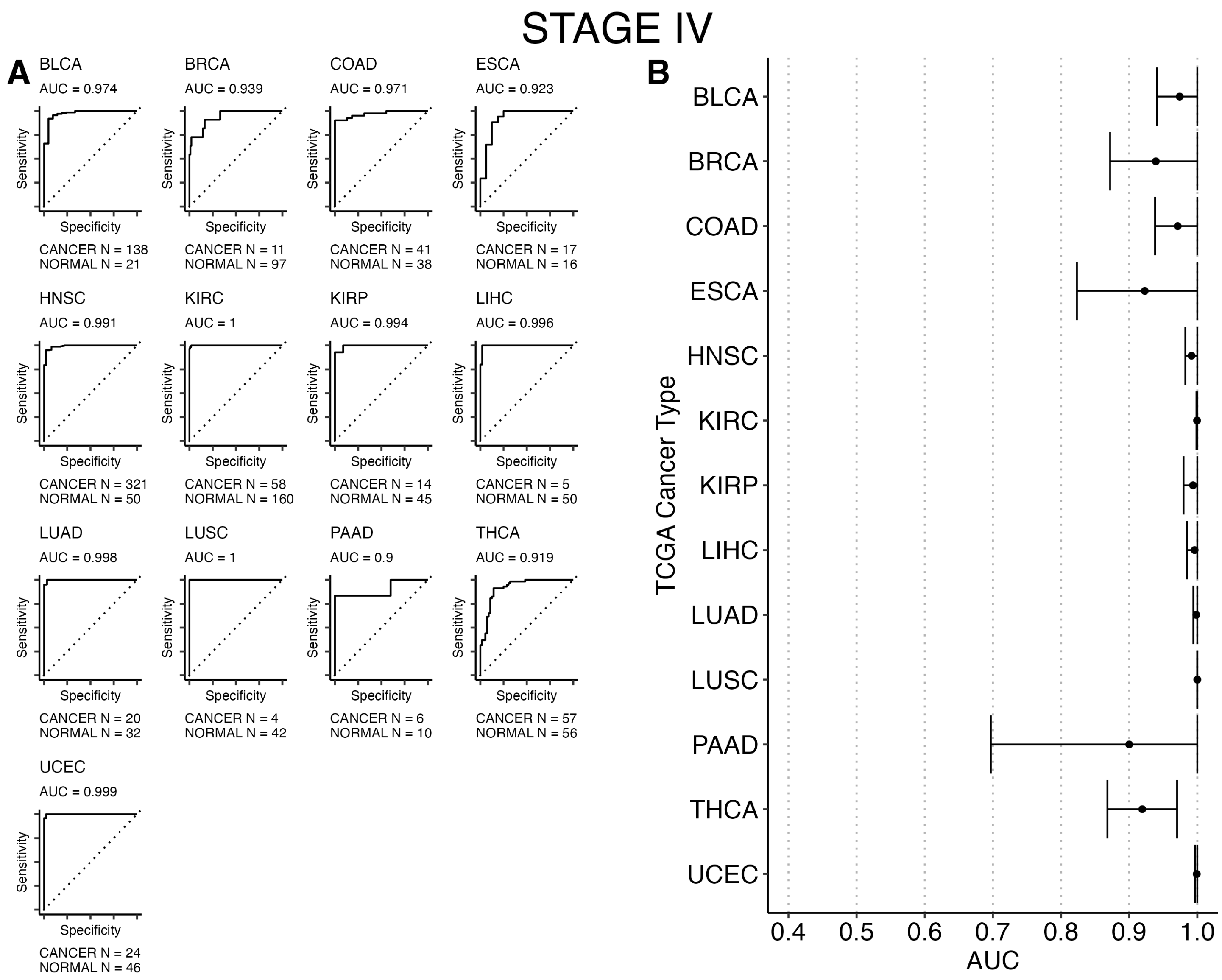

Supplemental Figure 7: mDMRs detected in multiple stage IV adult cancers from TCGA
(A) ROC curves from TCGA 450K DNA methylation data using a random forest model trained 422 of the 905 pediatric cancer mDMRs derived by WGBS (subset of 422 regions used due to the limitations of the 450K array). Plots annotated with TCGA cancer code and AUC. **(B)** Graphical representation of AUC in A with 95% CI indicated as error bars. See the GDC website for study abbreviation disambiguation (gdc.cancer.gov/resources-tcga-users/tcga-code-tables/tcga-study-abbreviations).

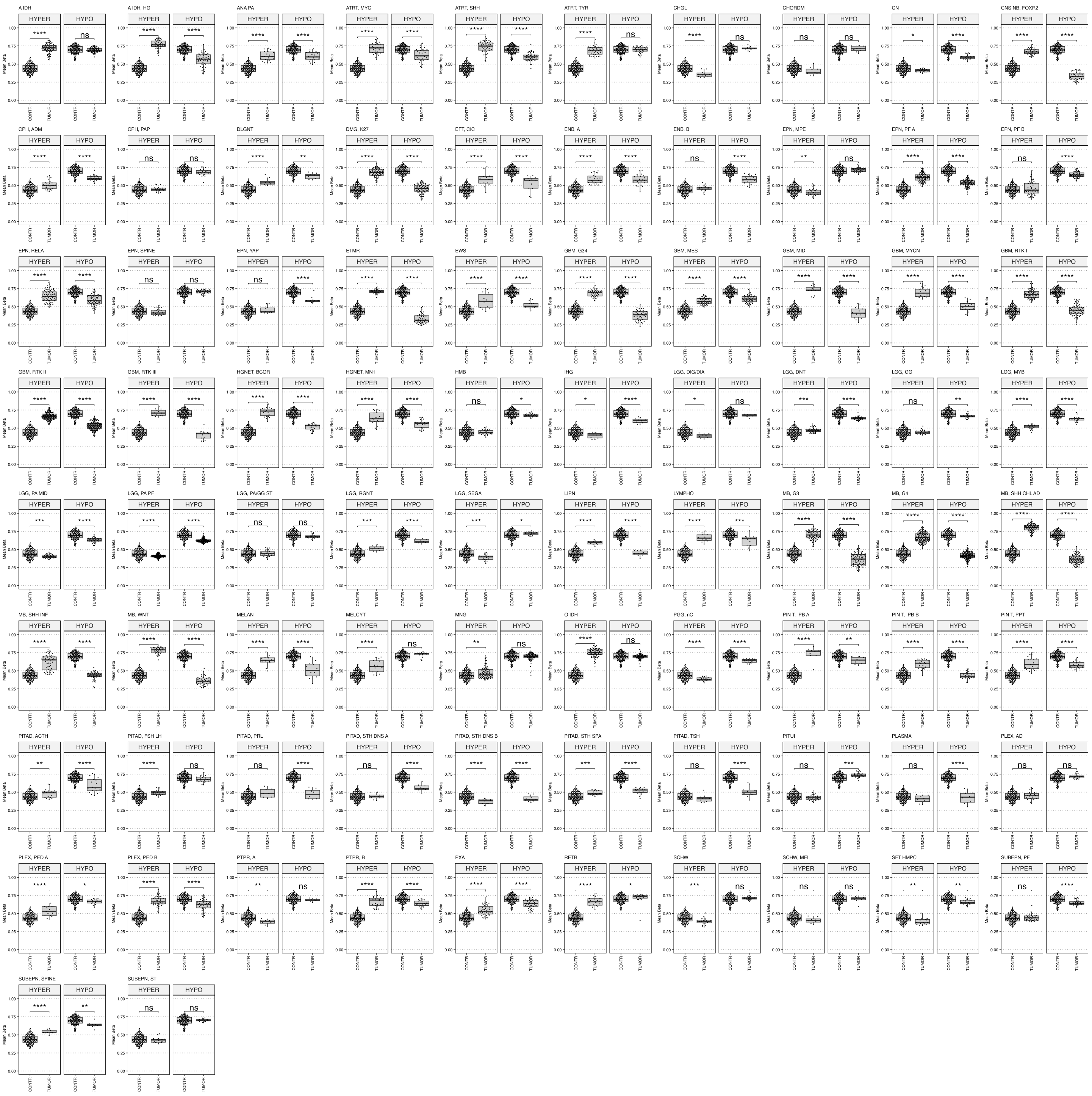

Supplemental Figure 8: mDMRs in CNS tumor (Capper et al.) dataset

Tumor and normal mean beta values across hypermethylated and hypomethylated mDMRs in CNS tumors. Each plot represents one methylation class from Capper et al.

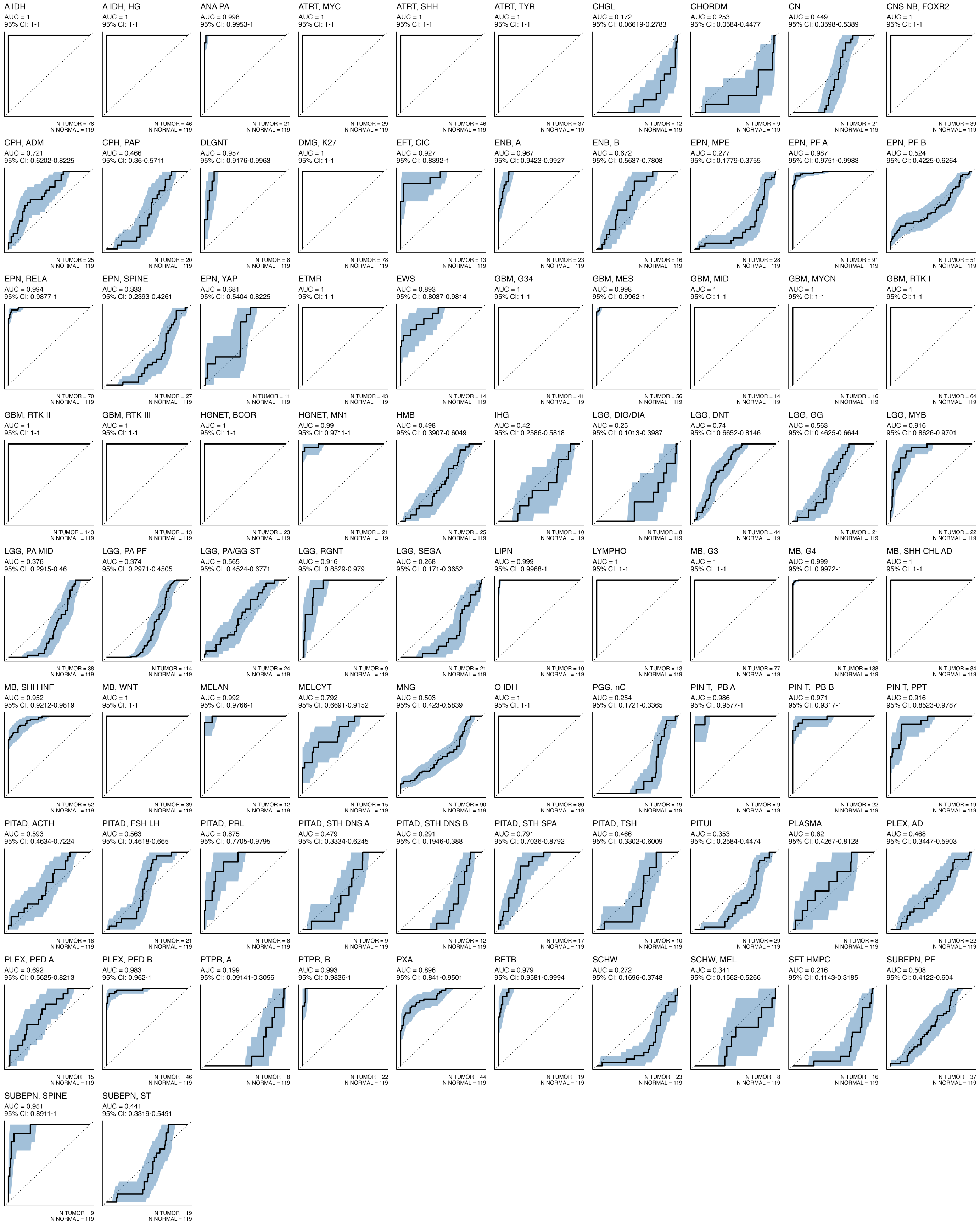

Supplemental Figure 9: ROC curves from mDMRs in CNS tumor (Capper et al.) dataset
ROC curves from Capper et al. 450K DNA methylation data using a random forest model trained 422 of the 905 pediatric cancer mDMRs derived by WGBS (subset of 422 regions used due to the limitations of the 450K array). Each plot represents one methylation class from Capper et al.

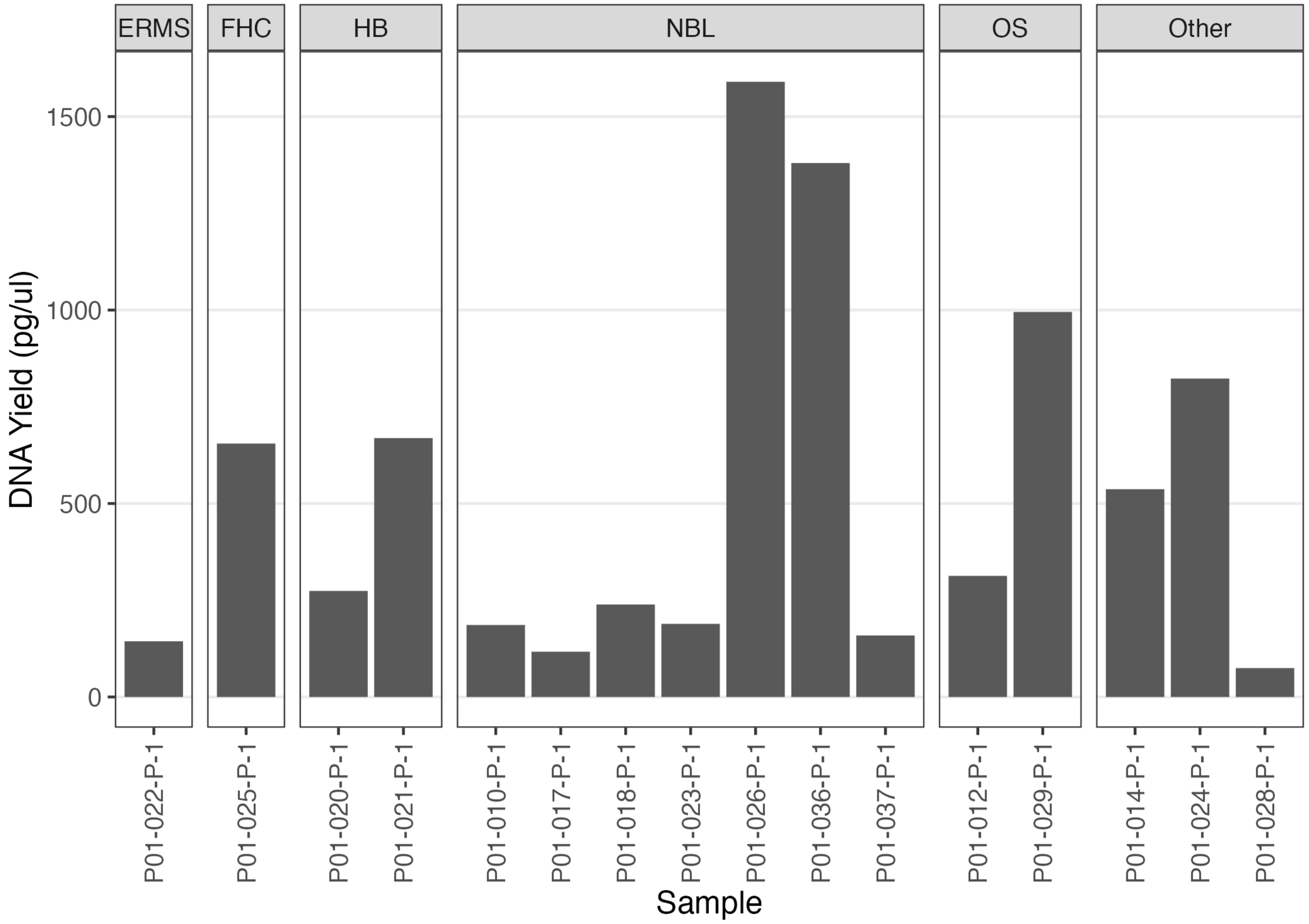

Supplemental Figure 10: Cell free DNA yield from plasma.
DNA yield in pg/ul per plasma sample. Vertically subdivided by diagnosis.

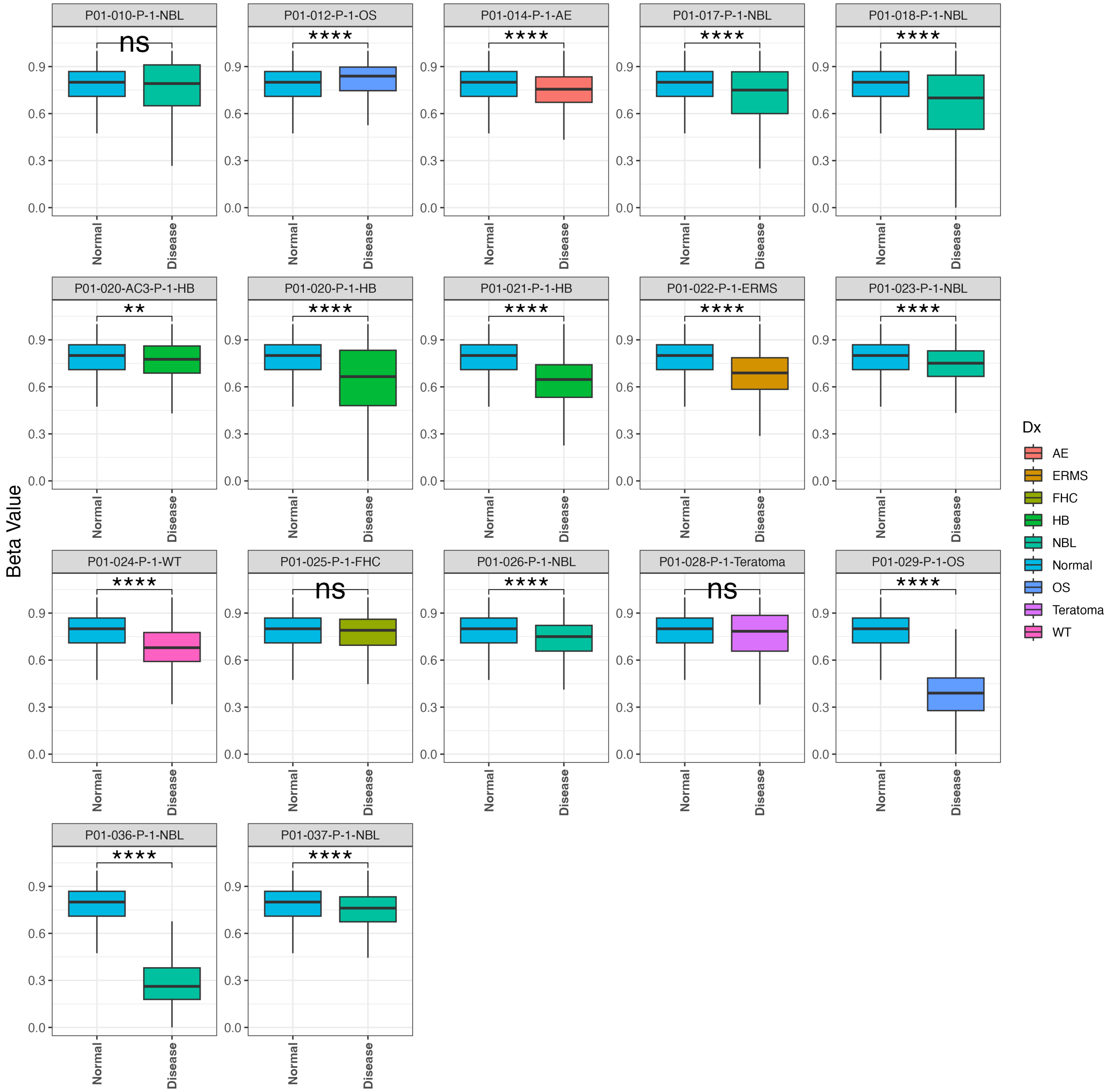

Supplemental Figure 11: Beta value within 402 hypomethylated mDMRs per sample.
Mean beta value across 402 mDMRs broken down by sample. Color indicates diagnosis, abbreviations are the same as in figure 1c.

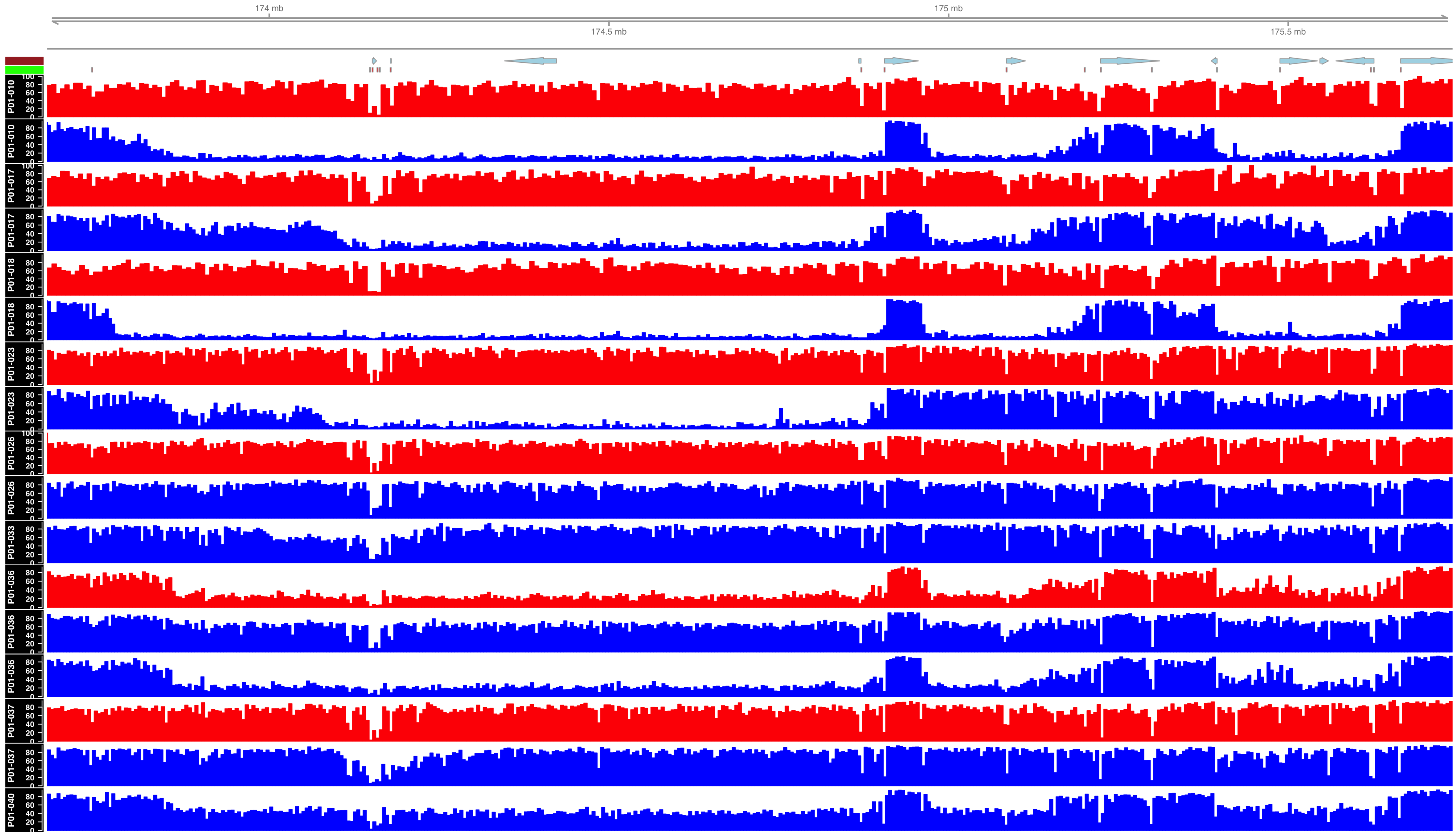

Supplemental Figure 12: Example of PMDs detected in cfDNA.

Example region spans 2.1Mb. Methylation tracks colored by sample type: blue = tissue, red = cfDNA. Annotated with chromosome ideogram track indicating the location of this region on chromosome 5, gene track (light blue arrows denote location and direction of genes) and CpG island track (red blocks).

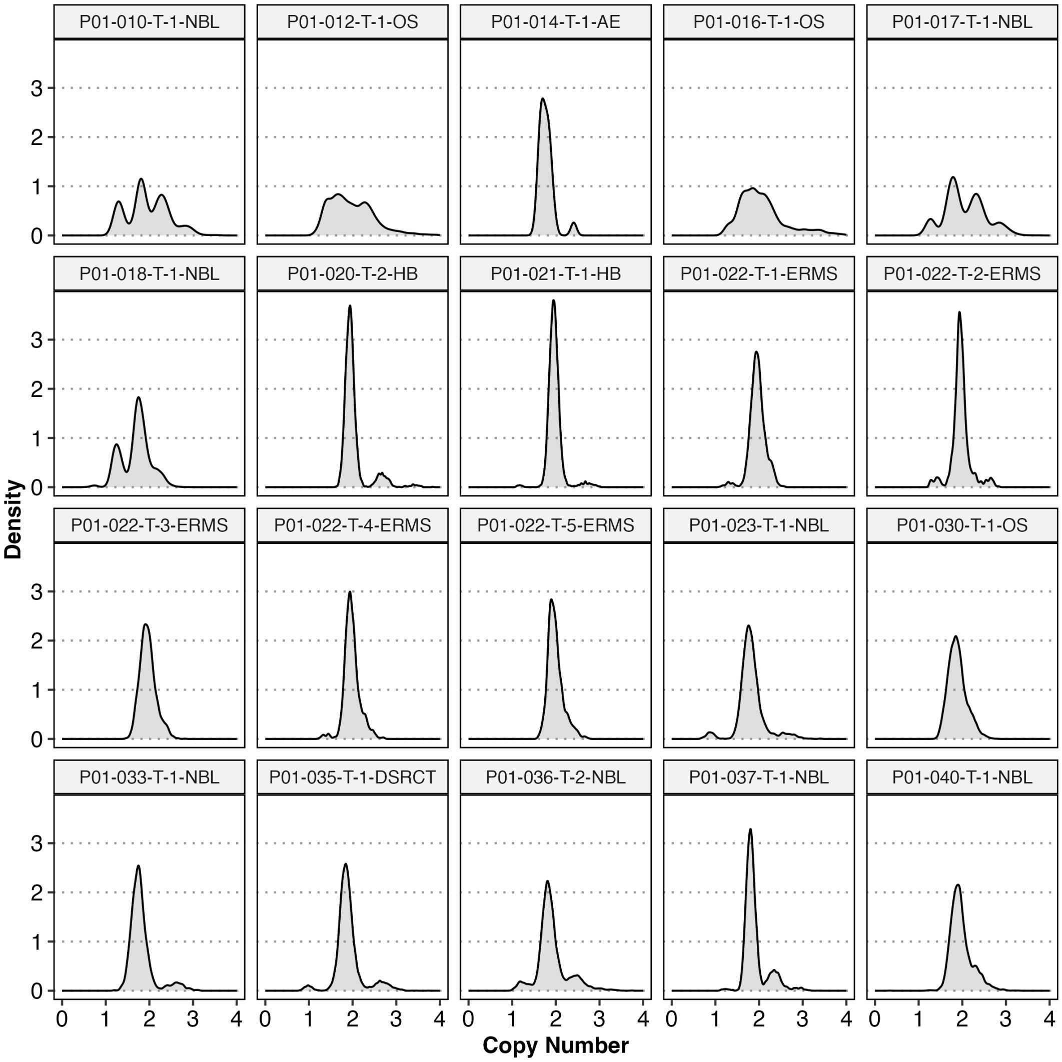

Supplemental Figure 13: Copy number profiles within PMD calls.
Density plots display the distribution of gDNA copy number within PMD calls and methylation deserts per sample. Plots do not show a consistent shift towards copy number loss or gain within PMDs or deserts. This observation is consistent with permutation testing which did not find significant associations between copy number alterations and PMDs or methylation deserts.

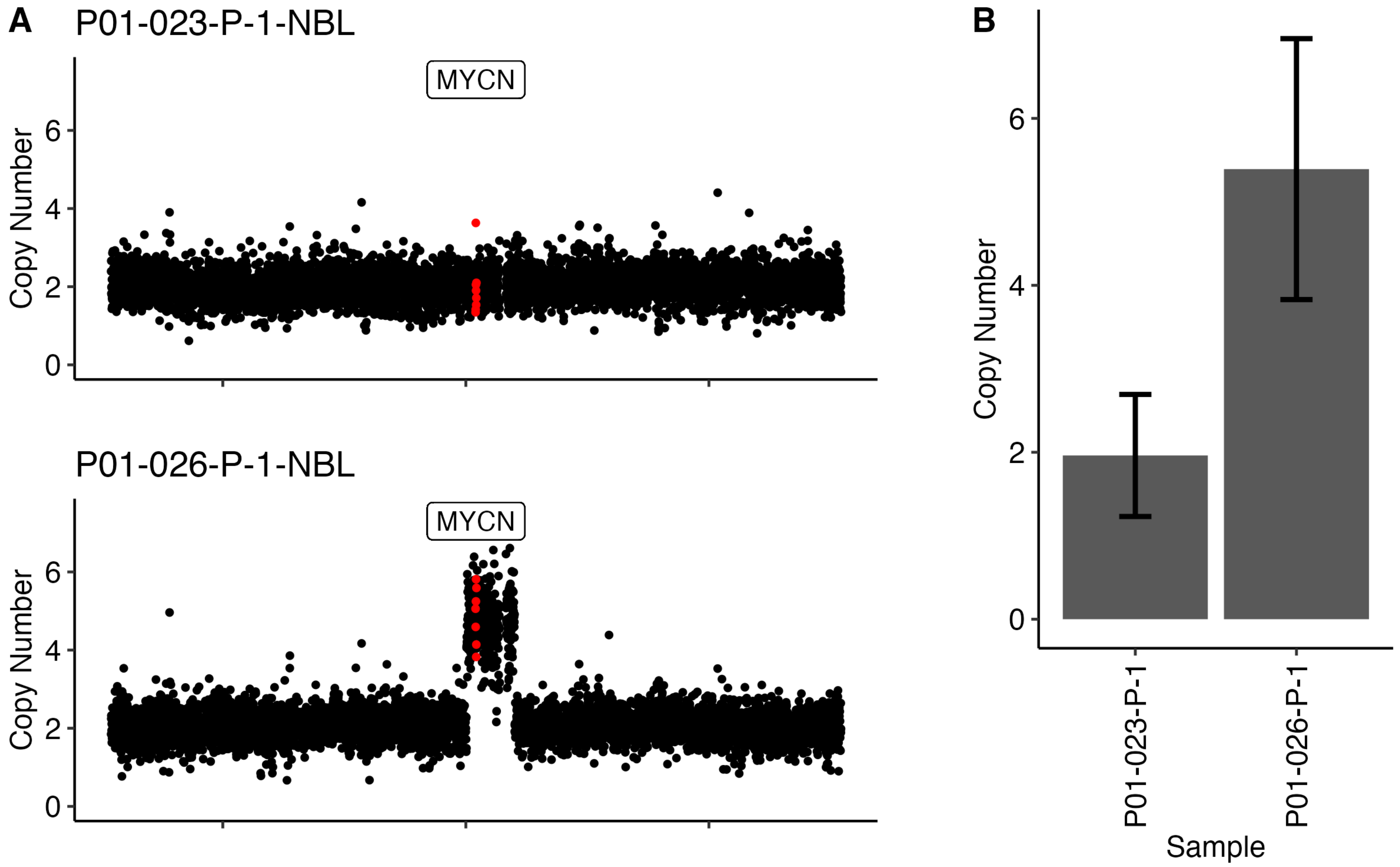

Supplemental Figure 14: MYCN amplification in cfDNA.
(A) Copy number plots of NBL samples which showed MYCN amplification (P01-023 & P01-026) in tumor tissue. MYCN location indicated by red points. Each point represents a 1kb region. **(B)** Average copy number of each 1kb bin across MYCN in aforementioned samples. Error bars represent ±1 standard deviation.

Dx	nCases	mean.nDMR	median.nDMR	min.nDMR	max.nDMR	mean.nCpG	median.width	CpG.Island.pct	CpG.Shore.pct	CpG.Shelf.pct	Open.Sea.pct
AE	1	146689	146689	146689	146689	13.55	645	7.75	10.65	4.71	76.88
DSRCT	1	296158	296158	296158	296158	15.55	899	4.04	7.56	3.45	84.96
ERMS	5	110497	110969	105219	115022	13.86	586	9.37	10.5	5.09	75.03
FHC	2	141053	141053	87056	195050	13.53	570.5	13.28	10.63	4.5	71.59
HB	3	181208	185167	127055	231401	13.54	641	6.26	10.46	4.87	78.41
Hodgkins	1	67071	67071	67071	67071	12.02	655	5.37	9.57	4.54	80.51
MRT	2	123592	123592	120378	126805	13.98	548	15.02	12.87	4.83	67.29
NBL	9	269160	269411	148053	363209	17.36	755	5.13	9.33	4.46	81.08
OS	4	324369	311860	204112	469644	15.65	775.5	4.61	6.46	3.78	85.15
Teratoma	2	196796	196796	110005	283588	14.26	633.5	5.76	9.31	4.76	80.16
WT	1	60101	60101	60101	60101	12.01	478	7.82	12.98	6.01	73.2

Supplemental Table 1: DMR summaries from tissue samples
The average, median, minimum, and maximum number of DMRs by cancer type. The number of cases for each type is also indicated. The average number of CpGs, median width for the DMRs, and locations with respect to CpG islands, shores, shelves and open sea are also indicated.

Canonical Pathways	p-value	Ratio	Molecules
NAD biosynthesis II (from tryptophan)	0	0.17	HAAO;NMNAT3
nNOS Signaling in Skeletal Muscle Cells	0	0.05	CACNA1I;CACNA2D4
Netrin Signaling	0	0.03	CACNA1I;CACNA2D4
FcγRIIB Signaling in B Lymphocytes	0	0.03	CACNA1I;CACNA2D4
CCR5 Signaling in Macrophages	0	0.02	CACNA1I;CACNA2D4
GABA Receptor Signaling	0	0.02	CACNA1I;CACNA2D4
GPCR-Mediated Nutrient Sensing in Enteroendocrine Cells	0	0.02	CACNA1I;CACNA2D4
NAD Biosynthesis from 2-amino-3-carboxymuconate Semialdehyde	0.01	0.17	NMNAT3
Tryptophan Degradation to 2-amino-3-carboxymuconate Semialdehyde	0.01	0.17	HAAO
NAD Biosynthesis III	0.01	0.17	NMNAT3
G Beta Gamma Signaling	0.01	0.02	CACNA1I;CACNA2D4
Endocannabinoid Neuronal Synapse Pathway	0.01	0.02	CACNA1I;CACNA2D4
White Adipose Tissue Browning Pathway	0.01	0.02	CACNA1I;CACNA2D4
Thioredoxin Pathway	0.01	0.14	TXNRD1
NAD Salvage Pathway III	0.01	0.14	NMNAT3
Androgen Signaling	0.01	0.01	CACNA1I;CACNA2D4
Type II Diabetes Mellitus Signaling	0.01	0.01	CACNA1I;CACNA2D4
Corticotropin Releasing Hormone Signaling	0.01	0.01	CACNA1I;CACNA2D4
PKCϑ Signaling in T Lymphocytes	0.01	0.01	CACNA1I;CACNA2D4
Gustation Pathway	0.01	0.01	CACNA1I;CACNA2D4
GNRH Signaling	0.01	0.01	CACNA1I;CACNA2D4
Synaptic Long Term Depression	0.01	0.01	CACNA1I;CACNA2D4
Calcium Signaling	0.02	0.01	CACNA1I;CACNA2D4
Role of NFAT in Cardiac Hypertrophy	0.02	0.01	CACNA1I;CACNA2D4
CREB Signaling in Neurons	0.02	0.01	CACNA1I;CACNA2D4;OXTR
Tryptophan Degradation III (Eukaryotic)	0.02	0.04	HAAO
Opioid Signaling Pathway	0.02	0.01	CACNA1I;CACNA2D4
Vitamin-C Transport	0.02	0.04	TXNRD1
NAD Salvage Pathway II	0.02	0.04	NMNAT3

Supplemental Table 2: IPA results from cluster 1 genes
Top canonical pathways associated with genes in cluster 1 in Figure 1A.

Canonical Pathways	p-value	Ratio	Molecules
Embryonic Stem Cell Differentiation into Cardiac Lineages	0.01	0.1	GATA4
Cardiomyocyte Differentiation via BMP Receptors	0.02	0.05	GATA4
Sonic Hedgehog Signaling	0.02	0.03	GLI3
Transcriptional Regulatory Network in Embryonic Stem Cells	0.04	0.02	GATA4

Supplemental Table 3: IPA results from cluster 2 genes
Top canonical pathways associated with genes in cluster 2 in Figure 1A.

Canonical Pathways	p-value	Ratio	Molecules
Gαs Signaling	0.01	0.02	PTGER4;SRC
Glycine Betaine Degradation	0.02	0.1	DMGDH
Ephrin Receptor Signaling	0.04	0.01	EFNA2;SRC
ERK/MAPK Signaling	0.05	0.01	SRC;VRK2

Supplemental Table 4: IPA results from cluster 3 genes
Top canonical pathways associated with genes in cluster 3 in Figure 1A.

Canonical Pathways	p-value	Ratio	Molecules
S-methyl-5'-thioadenosine Degradation II	0	0.33	MTAP
PI3K Signaling in B Lymphocytes	0.01	0.01	IRS2;NFATC1
Glycogen Degradation II	0.01	0.08	MTAP
DNA Double-Strand Break Repair by Homologous Recombination	0.01	0.07	BRCA1
Glycogen Degradation III	0.01	0.07	MTAP
Glutaryl-CoA Degradation	0.01	0.06	CDYL
GADD45 Signaling	0.01	0.05	BRCA1
DNA damage-induced 14-3-3σ Signaling	0.01	0.05	BRCA1
Tryptophan Degradation III (Eukaryotic)	0.02	0.04	CDYL
IL-9 Signaling	0.03	0.03	IRS2
Role of JAK2 in Hormone-like Cytokine Signaling	0.03	0.03	IRS2
April Mediated Signaling	0.03	0.03	NFATC1
B Cell Activating Factor Signaling	0.03	0.02	NFATC1
Role of Oct4 in Mammalian Embryonic Stem Cell Pluripotency	0.04	0.02	BRCA1
Cell Cycle: G2/M DNA Damage Checkpoint Regulation	0.04	0.02	BRCA1
Amyloid Processing	0.04	0.02	CDK5R1
Transcriptional Regulatory Network in Embryonic Stem Cells	0.04	0.02	CDYL
Role of CHK Proteins in Cell Cycle Checkpoint Control	0.04	0.02	BRCA1
Wnt/Ca+ pathway	0.05	0.02	NFATC1
Thrombopoietin Signaling	0.05	0.02	IRS2

Supplemental Table 5: IPA results from cluster 4 genes

Top canonical pathways associated with genes in cluster 4 in Figure 1A.

Dx	Primary Tumor	Recurrence	Solid Tissue Normal	Total
NBL	212	9	12	233
OS	86	0	0	86
MRT	68	0	0	68
WT	125	6	0	131
Total	491	15	12	518

Supplemental Table 6: TARGET sample summary
Table shows the number of samples for each cancer type accessed from the TARGET database.

tissue.type	n
adipose tissue	8
adrenal gland tissue	3
colon tissue	8
gastrointestinal tissue	18
liver tissue	1
lung tissue	4
muscle tissue	4
nervous tissue	4
pancreatic tissue	4
reproductive tissue	13
skin tissue	8
spleen tissue	3
thyroid gland tissue	4
vascular tissue	8
Total	90

Supplemental Table 7: ENCODE sample summary
Table shows the number of samples for each tissue type accessed from the ENCODE database.

Canonical Pathways	p-value	Ratio	Molecules
GABA Receptor Signaling	0	0.05	CACNA1A;CACNG6;GNA12;GNG7;ITPR2;KCNQ3
RHOGDI Signaling	0	0.03	ARHGAP8/PRR5-ARHGAP8;CDH4;GNA12;GNG7;ITGAD;ITGAX;MYH14
Androgen Signaling	0	0.04	CACNA1A;CACNG6;GNA12;GNG7;ITPR2;TAF2
GPCR-Mediated Nutrient Sensing in Enteroendocrine Cells	0	0.04	CACNA1A;CACNG6;GNG7;ITPR2;PYY
Phagosome Formation	0	0.02	ADGRB1;ADGRE3;AP1M2;FCER2;HRH2;ITGAD;ITGAX;ITPR2;MYH14;SSTR5;VAV1
G Beta Gamma Signaling	0	0.04	CACNA1A;CACNG6;GNA12;GNG7;ITPR2
CREB Signaling in Neurons	0	0.02	ADGRB1;ADGRE3;CACNA1A;CACNG6;GNA12;GNG7;HRH2;ITPR2;NTRK2;SSTR5
Cellular Effects of Sildenafil (Viagra)	0	0.03	CACNA1A;CACNG6;ITPR2;KCNQ3;MYH14
Crosstalk between Dendritic Cells and Natural Killer Cells	0	0.04	KIR2DL1/KIR2DL3;KIR3DL1;KIR3DL2;KIR3DL3
Cardiac β-adrenergic Signaling	0	0.03	CACNA1A;CACNG6;GNA12;GNG7;PDE6B
Signaling by Rho Family GTPases	0	0.02	CDH4;GNA12;GNG7;ITGAD;ITGAX;SEPTIN14
nNOS Signaling in Skeletal Muscle Cells	0	0.06	CACNA1A;CACNG6;ITPR2
Natural Killer Cell Signaling	0	0.03	KIR2DL1/KIR2DL3;KIR3DL1;KIR3DL2;KIR3DL3;VAV1
Cardiac Hypertrophy Signaling (Enhanced)	0	0.01	CACNA1A;CACNG6;GNA12;GNG7;ITGAD;ITGAX;ITPR2;PDE6B
Ephrin B Signaling	0	0.04	GNA12;GNG7;VAV1
Netrin Signaling	0	0.04	CACNA1A;CACNG6;ITPR2
Dilated Cardiomyopathy Signaling Pathway	0	0.03	CACNA1A;CACNG6;ITPR2;MYH14
GPCR-Mediated Integration of Enteroendocrine Signaling Exemplified by an L Cell	0	0.04	ITPR2;PYY;SSTR5
Endocannabinoid Neuronal Synapse Pathway	0	0.03	CACNA1A;CACNG6;GNG7;ITPR2
Actin Cytoskeleton Signaling	0	0.02	GNA12;ITGAD;ITGAX;MYH14;VAV1
TREM1 Signaling	0	0.04	ITGAX;NLRP12;NLRP9
Bupropion Degradation	0.01	0.08	CYP2B6;CYP2F1
Sperm Motility	0.01	0.02	GNA12;GNG7;ITPR2;NTRK2;STYK1
FcγRIIB Signaling in B Lymphocytes	0.01	0.04	CACNA1A;CACNG6;ITPR2
Opioid Signaling Pathway	0.01	0.02	CACNA1A;CACNG6;GNA12;GNG7;ITPR2
Actin Nucleation by ARP-WASP Complex	0.01	0.03	GNA12;ITGAD;ITGAX
Acetone Degradation I (to Methylglyoxal)	0.01	0.06	CYP2B6;CYP2F1
GNRH Signaling	0.01	0.02	CACNA1A;CACNG6;GNG7;ITPR2
Neuropathic Pain Signaling In Dorsal Horn Neurons	0.01	0.03	ITPR2;KCNQ3;NTRK2
Synaptic Long Term Depression	0.01	0.02	CACNA1A;CACNG6;GNA12;ITPR2
α-Adrenergic Signaling	0.01	0.03	GNA12;GNG7;ITPR2
Gustation Pathway	0.01	0.02	CACNA1A;CACNG6;ITPR2;KCNQ3
Ephrin Receptor Signaling	0.01	0.02	GNA12;GNG7;ITGAD;ITGAX
Sertoli Cell-Sertoli Cell Junction Signaling	0.01	0.02	ITGAD;ITGAX;SPTA1;TJP3
Calcium Signaling	0.02	0.02	CACNA1A;CACNG6;ITPR2;MYH14
Nitric Oxide Signaling in the Cardiovascular System	0.02	0.03	CACNA1A;CACNG6;ITPR2
G Protein Signaling Mediated by Tubby	0.02	0.05	GNA12;GNG7
Estrogen Biosynthesis	0.02	0.05	CYP2B6;CYP2F1
Role of NFAT in Cardiac Hypertrophy	0.02	0.02	CACNA1A;CACNG6;GNG7;ITPR2
Gαs Signaling	0.02	0.02	GNA12;GNG7;HRH2
RHOA Signaling	0.02	0.02	ARHGAP8/PRR5-ARHGAP8;GNA12;SEPTIN14
Apelin Muscle Signaling Pathway	0.02	0.04	GNA12;GNG7
fMLP Signaling in Neutrophils	0.02	0.02	GNA12;GNG7;ITPR2
Gα12/13 Signaling	0.02	0.02	CDH4;GNA12;VAV1
CCR3 Signaling in Eosinophils	0.02	0.02	GNA12;GNG7;ITPR2
Role of PKR in Interferon Induction and Antiviral Response	0.02	0.02	NLRP12;NLRP8;NLRP9
White Adipose Tissue Browning Pathway	0.02	0.02	CACNA1A;CACNG6;ITPR2
Oxidative Ethanol Degradation III	0.03	0.04	CYP2B6;CYP2F1
Nicotine Degradation III	0.03	0.03	CYP2B6;CYP2F1
Cardiac Hypertrophy Signaling	0.03	0.02	CACNA1A;CACNG6;GNA12;GNG7
Corticotropin Releasing Hormone Signaling	0.03	0.02	CACNA1A;CACNG6;ITPR2
Melatonin Degradation I	0.03	0.03	CYP2B6;CYP2F1
PTEN Signaling	0.03	0.02	ITGAD;ITGAX;NTRK2
Semaphorin Neuronal Repulsive Signaling Pathway	0.03	0.02	CRMP1;ITGAD;ITGAX
Type II Diabetes Mellitus Signaling	0.03	0.02	CACNA1A;CACNG6;ITPR2
Relaxin Signaling	0.03	0.02	GNA12;GNG7;PDE6B
Insulin Secretion Signaling Pathway	0.03	0.01	AGO2;CACNA1A;CACNG6;ITPR2
Superpathway of Melatonin Degradation	0.03	0.03	CYP2B6;CYP2F1
Nicotine Degradation II	0.03	0.03	CYP2B6;CYP2F1
Human Embryonic Stem Cell Pluripotency	0.04	0.02	GNA12;GNG7;NTRK2
CXCR4 Signaling	0.04	0.02	GNA12;GNG7;ITPR2
Gαq Signaling	0.04	0.02	GNA12;GNG7;ITPR2
Caveolar-mediated Endocytosis Signaling	0.04	0.03	ITGAD;ITGAX
Regulation of eIF4 and p70S6K Signaling	0.05	0.02	AGO2;ITGAD;ITGAX
Dopamine-DARPP32 Feedback in cAMP Signaling	0.05	0.02	CACNA1A;CACNG6;ITPR2
Maturity Onset Diabetes of Young (MODY) Signaling	0.05	0.03	CACNA1A;CACNG6

Supplemental Table 8: IPA results from hypomethylated mDMRs
Top canonical pathways associated with genes in hypomethylated mDMRs.

Canonical Pathways	p-value	Ratio	Molecules
IL-12 Signaling and Production in Macrophages	0	0.04	IL12RB2;MAPK13;MST1R;PIK3CD;PIK3R4;RXRA
Non-Small Cell Lung Cancer Signaling	0	0.05	CDK6;PIK3CD;PIK3R4;RASSF1;RXRA
CREB Signaling in Neurons	0	0.02	ADGRL4;ADRA1A;ADRA2A;ADRA2B;CACNA1I;CACNA2D4;GPR62;GRID1;LPAR5;NPFFR1;NTRK1;PDGFRA;PIK3CD;PIK3R4
Chronic Myeloid Leukemia Signaling	0	0.05	CDK6;CTBP2;PIK3CD;PIK3R4;STAT5A
Cardiac Hypertrophy Signaling	0	0.03	ADRA1A;ADRA2A;ADRA2B;CACNA2D4;MAPK13;PIK3CD;PIK3R4;RHOU
IL-9 Signaling	0	0.09	PIK3CD;PIK3R4;STAT5A
Role of Tissue Factor in Cancer	0.01	0.04	FGR;MAPK13;PIK3CD;PIK3R4;STAT5A
Human Embryonic Stem Cell Pluripotency	0.01	0.04	LEFTY1;LEFTY2;NTRK1;PDGFRA;PIK3CD;PIK3R4
IL-7 Signaling Pathway	0.01	0.05	MAPK13;PIK3CD;PIK3R4;STAT5A
IL-15 Production	0.01	0.04	FGR;MST1R;NTRK1;PDGFRA;ROR2
FLT3 Signaling in Hematopoietic Progenitor Cells	0.01	0.05	MAPK13;PIK3CD;PIK3R4;STAT5A
Reelin Signaling in Neurons	0.01	0.04	ARHGEF4;FGR;MAPK8IP2;PIK3CD;PIK3R4
FGF Signaling	0.01	0.05	FGF8;MAPK13;PIK3CD;PIK3R4
FcγRIIB Signaling in B Lymphocytes	0.01	0.05	CACNA1I;CACNA2D4;PIK3CD;PIK3R4
IL-23 Signaling Pathway	0.01	0.07	IL12RB2;PIK3CD;PIK3R4
AMPK Signaling	0.01	0.03	ADRA1A;ADRA2A;ADRA2B;CHRNE;MAPK13;PIK3CD;PIK3R4
White Adipose Tissue Browning Pathway	0.01	0.04	CACNA1I;CACNA2D4;CTBP2;MAPK13;RXRA
Regulation Of The Epithelial Mesenchymal Transition By Growth Factors Pathway	0.01	0.03	FGF8;MAPK13;MEST;PDGFRA;PIK3CD;PIK3R4
Xenobiotic Metabolism General Signaling Pathway	0.01	0.04	MAPK13;PIK3CD;PIK3R4;RXRA;SLC51A
UVB-Induced MAPK Signaling	0.02	0.06	MAPK13;PIK3CD;PIK3R4
Small Cell Lung Cancer Signaling	0.02	0.04	CDK6;PIK3CD;PIK3R4;RXRA
EGF Signaling	0.02	0.05	MAPK13;PIK3CD;PIK3R4
Insulin Secretion Signaling Pathway	0.02	0.03	CACNA1I;CACNA2D4;FGR;MAPK13;PIK3CD;PIK3R4;STAT5A
Role of IL-17A in Arthritis	0.02	0.05	MAPK13;PIK3CD;PIK3R4
MSP-RON Signaling Pathway	0.02	0.05	MST1R;PIK3CD;PIK3R4
eNOS Signaling	0.02	0.03	AQP2;CHRNE;LPAR5;PIK3CD;PIK3R4
IL-2 Signaling	0.02	0.05	PIK3CD;PIK3R4;STAT5A
IL-22 Signaling	0.02	0.08	MAPK13;STAT5A
Thrombopoietin Signaling	0.03	0.05	PIK3CD;PIK3R4;STAT5A
Role of JAK family kinases in IL-6-type Cytokine Signaling	0.03	0.08	MAPK13;STAT5A
ERB2-ERBB3 Signaling	0.03	0.05	PIK3CD;PIK3R4;STAT5A
Amyotrophic Lateral Sclerosis Signaling	0.03	0.03	CACNA2D4;GRID1;PIK3CD;PIK3R4
Glioblastoma Multiforme Signaling	0.03	0.03	CDK6;PDGFRA;PIK3CD;PIK3R4;RHOU
Superpathway of Inositol Phosphate Compounds	0.03	0.03	ACP5;ITPKA;PIK3CD;PIK3R4;PIP5K1C;PPFIA3
CD40 Signaling	0.03	0.04	MAPK13;PIK3CD;PIK3R4
IL-17A Signaling in Airway Cells	0.03	0.04	MAPK13;PIK3CD;PIK3R4
S-methyl-5'-thioadenosine Degradation II	0.03	0.33	MTAP
Sphingosine-1-phosphate Signaling	0.03	0.03	PDGFRA;PIK3CD;PIK3R4;RHOU
Role of JAK1 and JAK3 in γc Cytokine Signaling	0.03	0.04	PIK3CD;PIK3R4;STAT5A
Role of p14/p19ARF in Tumor Suppression	0.03	0.07	PIK3CD;PIK3R4
Growth Hormone Signaling	0.03	0.04	PIK3CD;PIK3R4;STAT5A
Glioma Signaling	0.04	0.03	CDK6;PDGFRA;PIK3CD;PIK3R4
Breast Cancer Regulation by Stathmin1	0.04	0.02	ADGRL4;ADRA1A;ADRA2A;ADRA2B;ARHGEF4;CDK6;GPR62;LPAR5;NPFFR1;PIK3CD;PIK3R4
Glioma Invasiveness Signaling	0.04	0.04	PIK3CD;PIK3R4;RHOU
Leptin Signaling in Obesity	0.04	0.04	PIK3CD;PIK3R4;POMC
Neurotrophin/TRK Signaling	0.04	0.04	NTRK1;PIK3CD;PIK3R4
Angiopoietin Signaling	0.04	0.04	PIK3CD;PIK3R4;STAT5A
Sperm Motility	0.04	0.02	CACNA1I;FGR;MST1R;NTRK1;PDGFRA;ROR2
Phagosome Formation	0.04	0.02	ADGRL4;ADRA1A;ADRA2A;ADRA2B;ELMO3;FGR;GPR62;LPAR5;NPFFR1;PIK3CD;PIK3R4;PIP5K1C
Gα12/13 Signaling	0.04	0.03	CDH23;LPAR5;PIK3CD;PIK3R4
Clathrin-mediated Endocytosis Signaling	0.04	0.03	DNM1;FGF8;PIK3CD;PIK3R4;PIP5K1C
IL-3 Signaling	0.04	0.04	PIK3CD;PIK3R4;STAT5A
Thyroid Cancer Signaling	0.04	0.04	NTRK1;PIK3CD;PIK3R4
Estrogen-Dependent Breast Cancer Signaling	0.04	0.04	PIK3CD;PIK3R4;STAT5A
STAT3 Pathway	0.05	0.03	IL12RB2;MAPK13;NTRK1;PDGFRA
Th2 Pathway	0.05	0.03	IL12RB2;PIK3CD;PIK3R4;STAT5A
JAK/STAT Signaling	0.05	0.04	PIK3CD;PIK3R4;STAT5A
RAC Signaling	0.05	0.03	ANK1;PIK3CD;PIK3R4;PIP5K1C

Supplemental Table 9: IPA results from hypermethylated mDMRs

Top canonical pathways associated with genes in hypermethylated mDMRs.

Tissue.Type	BLCA	BRCA	COAD	ESCA	HNSC	KIRC	KIRP	LIHC	LUAD	LUSC	PAAD	PRAD	THCA	UCEC	Total
Cancer	415	796	308	186	529	323	276	379	471	370	185	503	515	435	5691
Normal	21	97	38	16	50	160	45	50	32	42	10	50	56	46	713
Total	436	893	346	202	579	483	321	429	503	412	195	553	571	481	6404

Supplemental Table 10: TCGA sample summary

Table shows the number of samples for each cancer type (tumor and adjacent normal) accessed from the TCGA database.

Dx	nCases	mean.nDMR	median.nDMR	min.nDMR	max.nDMR	mean.nCpG	median.width	CpG.Island.pct	CpG.Shore.pct	CpG.Shelf.pct	Open.Sea.pct
AE	1	22949	22949	22949	22949	12.68	561	6.99	7.86	4.82	80.33
ERMS	1	77759	77759	77759	77759	13.35	740	3.04	6.17	4.35	86.44
FHC	1	16550	16550	16550	16550	13.16	432	12.04	13.96	5.79	68.22
HB	3	45276	49410	25063	61354	13.21	544	6.71	10.69	5.55	77.06
NBL	7	68396	42935	11088	280216	13.91	540	7.59	10.61	5.43	76.37
OS	2	167042	167042	12464	321619	15.15	679	7.97	8.84	4.93	78.26
Teratoma	1	16674	16674	16674	16674	12.46	411	11.83	10.9	5.23	72.04
WT	1	58224	58224	58224	58224	14.07	674	5.81	10.29	5.17	78.72

Supplemental Table 11: DMR summaries from plasma samples
The average, median, minimum, and maximum number of DMRs by cancer type. The number of cases for each type is also indicated. The average number of CpGs, median width for the DMRs, and locations with respect to CpG islands, shores, shelves and open sea are also indicated.

BSI.ID	Tumor.Type	Age.at.surgery	Sex
AAA000153	DSRCT	16	M
AAA010161	DSRCT	7	M
AAA011812	DSRCT	10	F
AAA011841	DSRCT	10	F
AAA012743	DSRCT	10	M
AAA009588	Neuroblastoma	0	M
AAA009596	Neuroblastoma	2	F
AAA009714	Neuroblastoma	10	M
AAA009727	Neuroblastoma	3	F
AAA009772	Neuroblastoma	6	F
AAA009923	Neuroblastoma	2	F
AAA009935	Neuroblastoma	2	F
AAA009955	Neuroblastoma	2	M
AAA009988	Neuroblastoma	3	M
AAA010103	Neuroblastoma	1	M
AAA002841	Osteosarcoma	14	M
AAA003165	Osteosarcoma	14	M
AAA009448	Osteosarcoma	16	M
AAA009738	Osteosarcoma	19	M
AAA009752	Osteosarcoma	16	M
AAA009886	Osteosarcoma	13	M
AAA010356	Osteosarcoma	11	M
AAA012396	Osteosarcoma	13	F
AAA013412	Osteosarcoma	8	F
AAA016160	Osteosarcoma	5	F
AAA004012	Rhabdomyosarcoma	13	M
AAA010286	Rhabdomyosarcoma	9	F
AAA010510	Rhabdomyosarcoma	5	M
AAA012345	Rhabdomyosarcoma	8	M
AAA002850	Teratoma	0	F
AAA002915	Teratoma	8	M
AAA004055	Teratoma	0	F
AAA005397	Teratoma	9	F
AAA015908	Teratoma	2	M
AAA002749	Wilms	3	F
AAA002750	Wilms	6	F
AAA002786	Wilms	1	F
AAA002795	Wilms	7	M
AAA002813	Wilms	3	M
AAA002822	Wilms	4	F
AAA002823	Wilms	5	M
AAA009770	Wilms	3	M
AAA009890	Wilms	3	M
AAA010032	Wilms	2	F

Supplemental Table 12: CHLA sample summary
All cases from CHLA with demographic information and cancer type.
